# Sec22b regulates phagosome maturation by promoting ORP8-mediated lipid exchange at endoplasmic reticulum-phagosome contact sites

**DOI:** 10.1038/s42003-023-05382-0

**Published:** 2023-10-04

**Authors:** Nina Criado Santos, Samuel Bouvet, Maria Cruz Cobo, Marion Mandavit, Flavien Bermont, Cyril Castelbou, Farah Mansour, Maral Azam, Francesca Giordano, Paula Nunes-Hasler

**Affiliations:** 1 Department of Pathology and Immunology, Geneva Center for Inflammation Research, Faculty of Medicine, University of Geneva, Centre Médicale Universitaire, 1 Rue Michel-Servet, Geneva, Switzerland; 2 Department of Cellular Physiology and Metabolism, Faculty of Medicine, University of Geneva, Centre Médicale Universitaire, 1 Rue Michel-Servet, Geneva, Switzerland; 3 Institute for Integrative Biology of the Cell (I2BC), CEA, CNRS, Université Paris-Saclay, Gif-sur-Yvette cedex, 91198 France; 4 Inserm U1280, Gif-sur-Yvette cedex, 91198 France

**Keywords:** Phagocytosis, Cellular imaging, Endoplasmic reticulum, SNARE, Lysosomes

## Abstract

Phagosome maturation is critical for immune defense, defining whether ingested material is destroyed or converted into antigens. Sec22b regulates phagosome maturation, yet how has remained unclear. Here we show Sec22b tethers endoplasmic reticulum-phagosome membrane contact sites (MCS) independently of the known tether STIM1. Sec22b knockdown increases calcium signaling, phagolysosome fusion and antigen degradation and alters phagosomal phospholipids PI(3)P, PS and PI(4)P. Levels of PI(4)P, a lysosome docking lipid, are rescued by Sec22b re-expression and by expression of the artificial tether MAPPER but not the MCS-disrupting mutant Sec22b-P33. Moreover, Sec22b co-precipitates with the PS/PI(4)P exchange protein ORP8. Wild-type, but not mutant ORP8 rescues phagosomal PI(4)P and reduces antigen degradation. Sec22b, MAPPER and ORP8 but not P33 or mutant-ORP8 restores phagolysosome fusion in knockdown cells. These findings clarify an alternative mechanism through which Sec22b controls phagosome maturation and beg a reassessment of the relative contribution of Sec22b-mediated fusion versus tethering to phagosome biology.

## Abstract (web summary)

Sec22b controls phagosome maturation by tethering ER-phagosome membrane contact sites independently of the tether STIM1.

## Introduction

Phagocytosis is a critical immune process through which phagocytic immune cells such as neutrophils, macrophages and dendritic cells (DCs) engulf foreign particles into a membrane-enclosed vacuole. The ingested material is either destroyed or processed into antigens, rendering phagocytosis important to both innate and adaptive immunity. Phagosomes are formed by actin-driven membrane remodeling, followed by pinching off and sequential maturation involving fusion with endosomes and lysosomes, which impart the phagosome with increasing degradative capabilities^1,2^. Endoplasmic reticulum (ER) membranes are also recruited to phagosomes, although the underlying mechanisms and their functional role have been debated^3,4^. In our previous research, we have found that the ER-resident calcium (Ca^2+^) regulator STIM1 drives ER recruitment to phagosomes in neutrophils, dendritic cells and phagocytic fibroblast models to form structures comprised of tightly tethered (10–30 nm) associations with the phagosomal membrane, called membrane contact sites (MCS)^5–8^. We found that MCS fostered localized calcium signals that promoted actin disassembly and lysosome fusion, driving phagocytic ingestion rates and maturation. However, even in *Stim1*^−/−^*Stim2*^−/−^ double knock-out cells, considerable STIM-protein independent MCS remained^6^, suggesting additional MCS tethers may regulate phagocytosis.

Sec22b is a multifunctional protein of the Soluble N-ethylmaleimide-Sensitive Factor Attachment Proteins Receptor (SNARE) family, generally associated with mediating membrane fusion, that localizes to both the ER and ER-to-Golgi intermediate compartment (ERGIC)^9,10^. Sec22b was originally described in the regulation of both retrograde and anterograde trafficking in the secretory pathway, in a role partially redundant with YKT6^9,11,12^. In addition to a short cytoplasmic C-terminus, a single transmembrane domain, and SNARE motif, it also possesses a large N-terminal longin domain that regulates its localization and function^10,13,14^. Notably, it was shown to tether the ER to the plasma membrane (PM) in bona fide MCS without mediating fusion due to its longin domain-mediated exclusion of SNAP-25 from SNARE complexes in neurons and Hela cells^14,15^. In macrophages, both positive and negative roles for Sec22b in controlling phagocytic rates were observed^16,17^, whereas, in DCs, Sec22b knockdown promoted phagosome maturation, increasing phagolysosome fusion without affecting phagocytic rates^18^. In all cases, Sec22b was proposed to achieve these effects by mediating the fusion of either ER or ERGIC membranes with the phagosome, though fusion per se was never assessed directly. In addition, it is unclear how the fusion of ER or ERGIC could reduce phagosome fusion with lysosomes.

In light of our observations of the intimate but non-fusogenic association of ER membranes with phagosomes in the context of STIM1-mediated MCS and studies showing the non-fusogenic tethering role of Sec22b at the PM, we sought to determine whether Sec22b controls phagocytosis or phagosome maturation through a tethering role at ER-phagosome contacts. To this end, we utilized a fibroblast cell line rendered phagocytic by ectopic expression of the IgG receptor FCGR2A, a cell model that is much more amenable to transfection of multiple fluorescent constructs and high-resolution imaging than natural phagocytes, and which we have extensively characterized and shown to recapitulate many key aspects of the phagocytic process^5,6^. By overexpressing and knocking down Sec22b in the presence and absence of STIM1, we show that Sec22b is present at and regulates ER-phagosome MCS independently of STIM proteins. Since modulation of Sec22b expression imparted only mild, both positive and negative roles on Ca^2+^ signaling, we further investigated whether Sec22b might instead regulate non-vesicular lipid transfer at these contacts since it is another function often associated with MCS that Sec22b was shown to regulate at the PM^8,14,15^. We provide evidence that Sec22b regulates phagosomal levels of phospholipids at least in part by recruiting the lipid transfer protein ORP8 (also called Osbpl8) and that this, in turn, controls phagolysosome fusion and phagosomal antigen degradation.

## Results

### Sec22b localizes to and modulates ER-Phagosome MCS

**Fig. 1:**
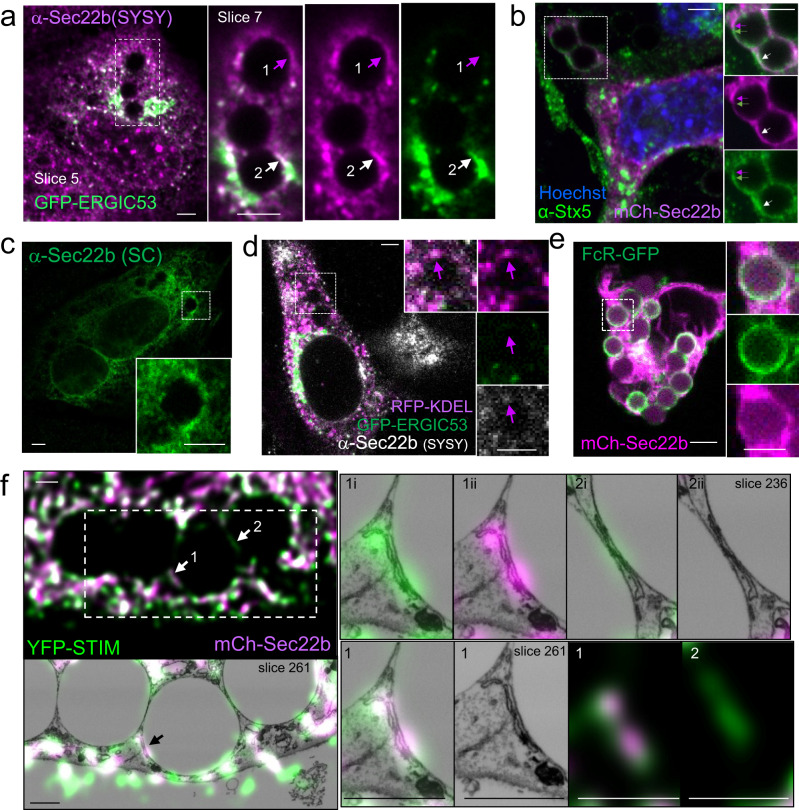
Sec22b localizes to ER-phagosome membrane contact sites. **a**. Synaptic Systems (SYSY) antibody staining of endogenous Sec22b (**a**, magenta) in phagocytic MEFs overexpressing GFP-ERGIC-53 (green). Sec22b periphagosomal accumulation devoid of (arrow 1) and containing (arrow 2) the ERGIC marker. Slice 5 of the confocal stack highlights the overall cellular staining, and Slice 7 in the inset highlights periphagosomal accumulation. **b** Immunostaining of endogenous ERGIC marker Stx5 (green) in phagocytic MEFs expressing mCherry (mCh) Sec22b (magenta). Arrows: periphagosomal punta containing Stx5 (green arrow), Sec22b (magenta arrow) or both (white arrow). **c** Immunostaining of endogenous Sec22b (green) in phagocytic MEFs using the Santa Cruz antibody (SC). **d** Immunostaining of endogenous Sec22b (white) in phagocytic MEFs overexpressing both the ER-marker RFP-KDEL (magenta) and GFP-ERGIC-53 (green). Arrow: periphagosomal accumulations of ER marker that are devoid of ERGIC marker and contain Sec22b. **e** MEFs overexpressing FCGR2A-GFP (FcR, green) and mCh-Sec22b (magenta). **f** 3D-CLEM analysis of phagocytic *Stim1*^−/−^*;Stim2*^−/−^ MEFs overexpressing mCh-Sec22b (magenta) and YFP-STIM1 (green). Arrow 1: periphagosomal accumulation of Sec22b co-localizing with STIM1 in an MCS structure. Arrow 2: MCS containing only STIM1 but not Sec22b. One deconvolved confocal slice = ∽75 EM slices. Slice 261 of the EM stack is shown in the large images and insets of site 1, while slice 236 is shown for site 2. Insets labeled (i) show an overlay of the EM with the green channel and those labeled (ii) with the magenta channel. Cells were exposed to IgG beads for 30 min in (**a**–**d**) and (**f**) for 15 min in (**e**). For (**a**–**e**), bars = 3 µm; for (**f**), bars = 1 µm.

Since Sec22b tethers the ER and PM^15^, we first investigated whether Sec22b localizes to ER-phagosome contact sites (ER-Phg MCS). Sec22b localizes to both the ER and ERGIC in various cell types, including neurons and DCs, with an estimated 50:50 distribution in yeast and HEK293T and a visibly greater but unquantified proportion in the ERGIC in other cells^15,18–20^. Mouse embryonic fibroblasts (MEFs) rendered phagocytic by overexpression of myc-FCGR2A were used here as a phagocytic model that is genetically tractable and whose ER-Phg MCS we have characterized extensively. Immunostainings with two commercial antibodies against Sec22b (SC: Santa Cruz Biotechnologies sc-101267 and SYSY: Synaptic Systems 186003) were performed in phagocytic MEFs exposed to IgG-coated targets. Both antibodies showed staining of variable quality but were consistent with both ER and ERGIC localization, although the SC antibody showed a larger ER pool, while the SYSY antibody preferentially highlighted ERGIC structures (Fig. 1a–c). Nevertheless, endogenous Sec22b immunostainings with the SYSY antibody, whose specificity we validated in Sec22b knockdown cells (see below and Supplementary Fig. S1a), under co-transfection with the ERGIC marker GFP-ERGIC-53 (also called LMAN1, Fig. 1a) or both GFP-ERGIC-53 and KDEL-tagged, ER-targeted-RFP (RFP-KDEL) (Fig. 1d) displayed punctate periphagosomal Sec22b accumulations reminiscent of contact sites that did not co-localize with the ERGIC marker (Fig. 1a, Site 1), in addition to areas that did (Fig. 1a, Site 2). We also assessed whether periphagosomal Sec22b co-localizes with syntaxin 5 (Stx5), Sec22b’s cognate SNARE that localizes predominantly to the ERGIC compartment^21^. A similar pattern to GFP-ERGIC-53/anti-Sec22b co-staining was observed when cells were labeled with anti-Sec22b(SC) and anti-Stx5, where some puncta co-localized and others did not (Supplementary Fig. S1b). To confirm the heterogeneous nature of periphagosomal Sec22b puncta, mCherry-tagged Sec22b (mCh-Sec22b) was co-expressed with GFP-ERGIC-53 and co-stained with anti-Stx5, and a similar pattern was again observed (Fig. 1b, Supplementary Fig. S1c, d). Since the Fc-receptor stains the phagosomal membrane itself, co-expression of mCh-Sec22b with FCGR2A-GFP or of EGFP-Sec22b with myc-FCGR2A was also analyzed. The Fc-receptor displayed a thin continuous phagosomal staining that was distinct from the punctate nature of the Sec22b periphagosomal localization, suggesting that periphagosomal Sec22b puncta are likely close to but distinct from the phagosomal membrane (Fig. 1e, Supplementary Fig. S1e).

We have previously shown that the ER-resident Ca^2+^ regulator STIM1 localizes to ER-Phg MCS^5,7^. To visualize bona fide ER-Phg MCS, phagocytic *Stim1*^−/−^*/Stim2*^−/−^ double knock-out MEFs co-transfected with YFP-STIM1 and mCh-Sec22b were exposed to IgG-coated targets and analyzed by 3D correlation light electron microscopy (3D-CLEM). *Stim1*^−/−^*/Stim2*^−/−^ cells were employed to mitigate the effects of STIM1 overexpression^22^. Periphagosomal Sec22b puncta co-localized extensively with STIM1 (Fig. 1f, Site 1). 3D-CLEM not only confirmed that these structures did indeed correspond to bona fide MCS, but it also allowed a complete inspection of structures above and below the MCS that could contribute to a colocalization signal. In the structures indicated by the arrows (Fig. 1f, Site 1; Supplementary Fig. S1f), only other MCS and no other vesicular structures that could correspond to endosomes or ERGIC vesicles were close enough above or below the ER contacting the phagosome to contribute a signal (Supplementary Fig. S1f), providing strong evidence that Sec22b within the ER was indeed localizing to ER-Phg MCS. While most (though not all) periphagosomal structures containing Sec22b also contained STIM1, some periphagosomal MCS marked by STIM1 that were devoid of Sec22b were captured in the CLEM image (Fig. 1f, Site 2). This indicates that inclusion in an ER-Phg MCS is selective and not an automatic consequence of Sec22b’s overexpression within the ER or its co-expression with STIM1, whose overexpression causes an increase in the frequency and size of MCS^5^. Furthermore, it suggests that similar to ER-PM MCS, phagosomal MCS are also heterogeneous with a composition that differs not only from bulk ER but also between different contacts^8,23^.

**Fig. 2:**
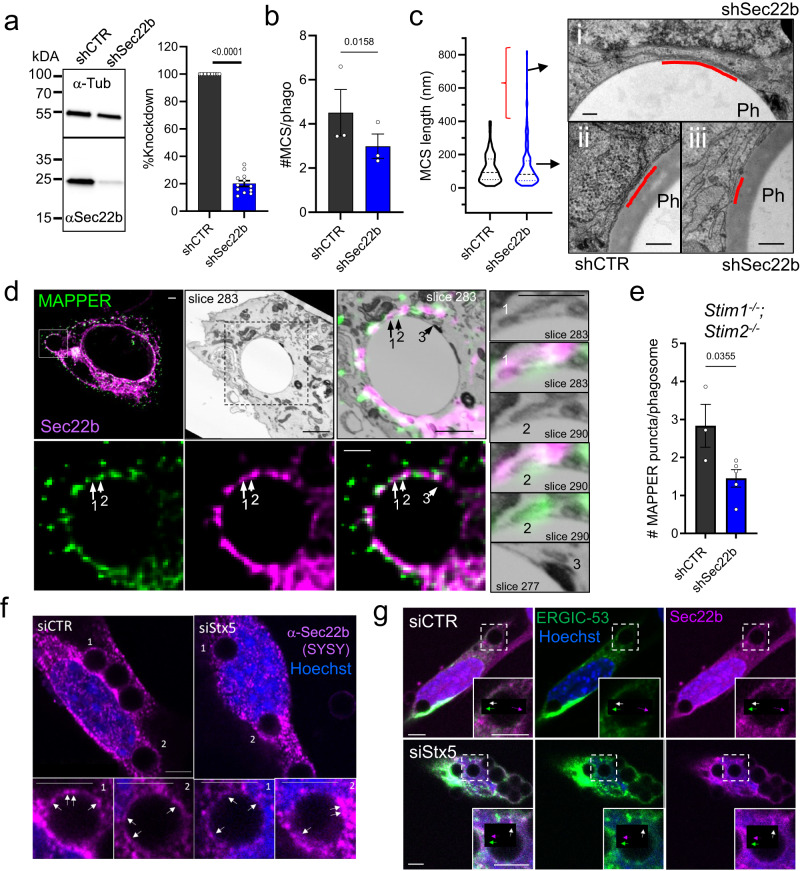
Sec22b localizes to phagosomal contact sites independently of STIM1 and Stx5. **a** Western blot of stable MEF cell lines expressing control (shCTR) or anti-Sec22b shRNA (shSec22b) (*n* = 12 biologically independent samples). **b** Quantification of ER-Phg MCS frequency in transmission EM slices (*n* = 3 biologically independent samples, 44/45 phagosomes shCTR/shSec22b). **c** Quantification of individual ER-Phg MCS length showing greater diversity in MCS length in shSec22b cells (F-test *p* < 0.0001). The small (3%) population of very large contacts in shSec22b cells is highlighted by the red bracket, an example is shown in panel (i). Typical examples of median MCS length are shown in panels (ii) and (iii). (*n* = 3 biologically independent samples, 172/145 contacts in 44/45 phagosomes shCTR/shSec22b). Bars = 100 nm. **d** 3D-CLEM analysis of *Stim1*^−/−^*;Stim2*^−/−^ phagocytic MEFs overexpressing EGFP-MAPPER (green) and mCh-Sec22b (magenta). Arrow 1: Sec22b-positive MCS (slice 283, insets). Arrow 2: MAPPER-positive MCS (slice 290, insets), Arrow 3: MCS devoid of both markers (slice 277, inset). Bars = 1 µm. **e** Quantification of periphagosomal EGFP-MAPPER puncta in *Stim1*^−/−^*;Stim2*^−/−^ cells stably transfected with shCTR or shSec22b (*n* = 3/5 biologically independent samples, 134/233 phagosomes in shCTR/shSec22b). Means ± SEM. **f** Immunostaining of endogenous Sec22b (SYSY antibody, magenta) in MEFs transfected with siCTR and siStx5 (10 nM). Arrows: periphagosomal Sec22b accumulations. Bars = 3 µm. (See also Supplementary Fig. S2) **g** Phagocytic MEFs co-transfected with siCTR or siStx5 and GFP-ERGIC-53 (green) and mCh-Sec22b (magenta). Arrows: periphagosomal punta containing ERGIC-53 (green arrow), Sec22b (magenta arrow) or both (white arrow). Bars = 3 µm.

To determine whether Sec22b contributes to ER-Phg MCS tethering, the effect of Sec22b knockdown on the frequency of periphagosomal MCS was assessed. Stable cell lines established by lentiviral transduction of control (shCTR) or short hairpin RNA (shRNA) targeting Sec22b (shSec22b) showed a knockdown efficiency of ⁓80% by Western blot and was confirmed by immunofluorescence (Fig. 2a, Supplementary Figs. S1a, S3a, b). Cells were then exposed to IgG-coupled targets, fixed and embedded for transmission electron microscopy (TEM)^5^. Quantification of periphagosomal MCS by morphological assessment of random TEM slices^5^ showed a ⁓30% loss in the frequency of periphagosomal MCS, from an average of 4.5 contacts per phagosome in shCTR cells to 3.0 in shSec22b cells (Fig. 2b, c). Whereas the median size of individual contacts was slightly lower (93 vs 81 nm in shCTR vs shSec22b, Fig. 2c), a small population (3%) of very large (>400 nm) contacts were observed uniquely in shSec22b cells (Fig. 2c, red bracket) resulting in an average size that was not significantly different. Recruitment of Sec22b was independent of STIM proteins since Sec22b still localized to ER-Phg MCS, as indicated by CLEM analysis, in phagocytic *Stim1*^−/−^*/Stim2*^−/−^ cells co-expressing mCh-Sec22b and the artificial MCS marker EGFP-MAPPER which labels MCS but does not majorly affect Ca^2+^ signaling^24^. In contrast to STIM1, Sec22b and MAPPER colocalization were less pronounced and appeared to segregate to adjacent MCS zones (Fig. 2d, sites 1 and 2). MCS devoid of either Sec22b or MAPPER were also observed (Fig. 2d, Site 3), again highlighting the heterogeneity of ER-Phg MCS. In addition, loss of Sec22b in *Stim1*^−/−^*/Stim2*^−/−^ cells led to a ⁓50% loss of MAPPER recruitment to phagosomes (Fig. 2e).

Furthermore, to gain insight into whether Stx5 could be involved in Sec22b recruitment to phagosomes, phagocytic MEFs were transfected with an siRNA pool directed against Stx5 (siStx5) that led to a reduction of ~65% of both the long and short isoforms of Stx5^21^ (Supplementary Fig. S2a, b). Phagosomal recruitment of Sec22b was then assessed. However, global levels of Sec22b immunostaining appeared lowered in siStx5-treated cells (Fig. 2f, Supplementary Fig. S2c), which by Western blot was estimated to represent a ~23% reduction (Supplementary Fig. S2a–c). Nevertheless, both endogenous (Fig. 2f) and well as overexpressed mCh-Sec22b (Supplementary Fig. S2d) periphagosomal puncta were still readily observed in siStx5 cells as compared to controls (siCTR). Moreover, GFP-ERGIC-53/mCh-Sec22b-positive periphagosomal structures were also readily observed, even in knockdown cells. Although we cannot exclude that residual Stx5 activity was sufficient to mediate Sec22b phagosomal recruitment, the data suggest that neither Stx5 nor STIM1 is strictly required for Sec22b recruitment to phagosomes. Regardless of the mechanism driving Sec22b to phagosomes, these data nevertheless demonstrate that ER-bound Sec22b localizes to and influences the frequency of ER-Phg MCS, supporting the view that ER-localized Sec22b may play a role during phagocytosis just as it does at the plasma membrane^14,15^.

### Sec22b is not required but can modulate Ca^2+^ signaling

**Fig. 3:**
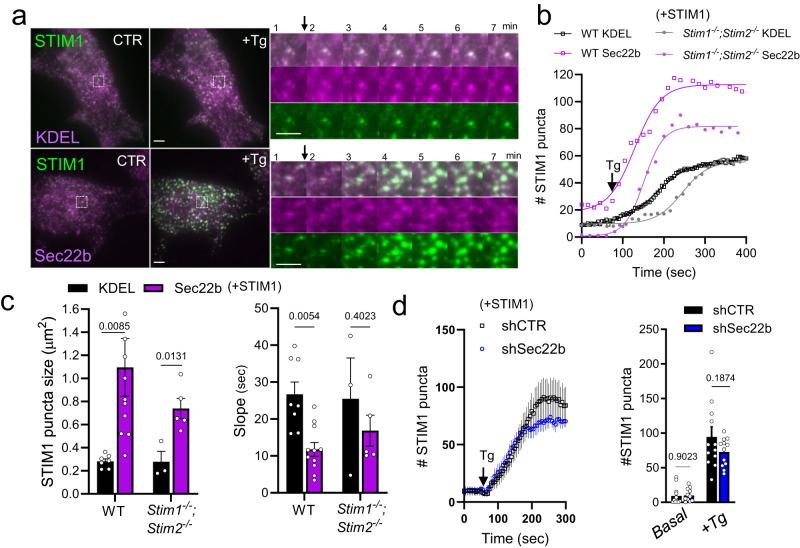
Sec22b facilitates recruitment of STIM1 to contact sites. **a**–**c** TIRF analysis of wild-type (WT) and *Stim1*^−/−^*;Stim2*^−/−^ MEFs transfected with YFP-STIM1 (green) in combination with mCh-Sec22b or RFP-KDEL controls (magenta) treated with 1 µM thapsigargin (Tg). **a** Panels labeled CTR are *t* = 0 min +Tg is the same cell at *t* = 7 min. Insets show a zoom of the timelapse every 1 min. Quantification of the number (**b**; see also Supplementary Fig. S3c), mean puncta size and kinetics (Slope, **c**) of STIM1 puncta 6 min after Tg addition. (*n* = 8/11;3/5 biologically independent samples WT;*Stim1*^−/−^*;Stim2*^−/−^ KDEL/Sec22b, 14/15; 9/13 cells). In (**b**), mean values and Boltzmann sigmoidal curve fits used to calculate slope parameters are shown. **d** Similar TIRF analysis as in (**a**–**c**) performed in shCTR and shSec22b *Stim1*^−/−^*;Stim2*^−/−^ cells (*n* = 13/12 biologically independent samples shCTR/shSec22b, 22/19 cells; see also Supplementary Fig. S3d). Error bars are omitted from traces in (**b**) for clarity.

The Ca^2+^ regulators STIM1 and its close homolog STIM2 function both at ER-PM MCS, promoting global Ca^2+^ signals, as well as at ER-Phg MCS, where particularly STIM1 plays a major role in generating localized Ca^2+^ hotspots^5,6^. Given the extensive colocalization of STIM1 and Sec22b, we next investigated whether Sec22b cooperates with STIM1 at MCS to regulate Ca^2+^ signaling. Total internal reflection microscopy (TIRF) analysis allowed simultaneous imaging of Sec22b and STIM1 recruitment to ER-PM MCS puncta during the activation of store-operated Ca^2+^ entry (SOCE) (Fig. 3a). Targeted activation of SOCE that does not rely on receptor-mediated lipid signaling is achieved by applying the sarcoplasmic reticulum ATPase inhibitor thapsigargin (Tg) which releases Ca^2+^ stored within the ER. This event promotes STIM1 activation, oligomerization and translocation to ER-PM MCS, steps which are necessary for STIM1 interaction with partner channels of the ORAI and TRPC families, initiating Ca^2+^ influx^25^. In order to determine whether Sec22b could influence STIM1 recruitment to ER-PM MCS, Tg was applied to either WT or *Stim1*^−/−^*/Stim2*^−/−^ MEFs overexpressing YFP-STIM1 and mCh-Sec22b or RFP-KDEL as control, in the absence of external Ca^2+^ (which prevents the negative feedback of STIM1 de-activation upon Ca^2+^ influx)^26^. As above, *Stim1*^−/−^*/Stim2*^−/−^ cells were employed to mitigate the effects of STIM1 overexpression, which, when excessive, can be saturating and inhibitory to Ca^2+^ signaling^22,25,26^. TIRF plane puncta, representing ER-PM MCS, increased in number and size following a sigmoidal time function (Fig. 3a, b, Supplementary Fig. S3a–c). Puncta were larger in both WT and *Stim1*^−/−^*/Stim2*^−/−^ cells (Fig. 3a–c) and more numerous (Supplementary Fig. S3c) in WT cells overexpressing Sec22b as compared to KDEL controls. In addition, STIM1 recruitment was accelerated, manifesting as either a decreased slope parameter in the sigmoidal curve fit in WT cells or a decreased time constant (t_50%_) in *Stim1*^−/−^*/Stim2*^−/−^ cells (Fig. 3c, Supplementary Fig. S3c). On the other hand, Sec22b knockdown had no significant effect on the kinetics of YFP-STIM1 recruitment (Fig. 3d, Supplementary Fig. S3a, b, d).

**Fig. 4:**
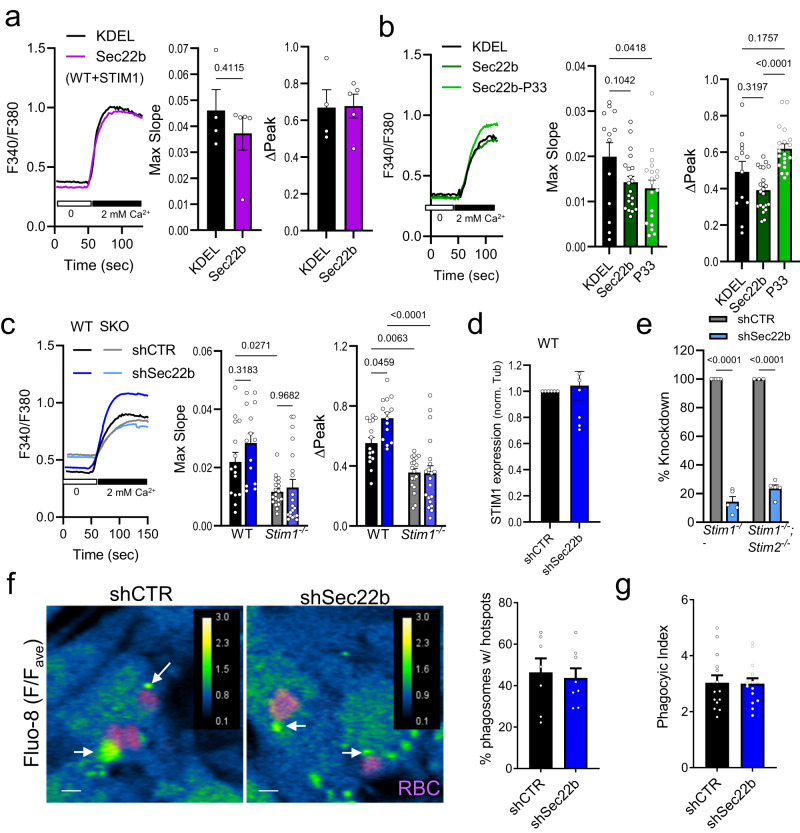
Sec22b affects global but not local calcium signaling. **a**–**c** Fura-2 analysis (F340/F380 ratio) of store-operated calcium influx after 8 min of 1 µM Tg/2 mM Ca^2+^ re-addition in **a** WT MEFs transfected with YFP-STIM1 and mCh-Sec22b or RFP-KDEL controls (*n* = 4/5 biologically independent samples; 124/163 cells KDEL/Sec22b), in **b** WT MEFs transfected only with GFP-KDEL, EGFP-Sec22b, or EGFP-Sec22b-P33 (*n* = 13/20/19 biologically independent samples, 124/217/117 cells KDEL/Sec22b/P33) and in **c** WT and *Stim1*^−/−^ shCTR and shSec22b stable cell lines (*n* = 15/13;19/20 biologically independent samples; 537/408;702/484 cells WT;*Stim1*^−/−^ shCTR/shSec22b). **d** Quantification of STIM1 expression by Western blot in WT MEF shCTR and shSec22b (*n* = 8 biologically independent samples; see also Supplementary Fig. S3b). **e** Quantification of percent Sec22b knockdown by Western blot in *Stim1*^−/−^ and *Stim1*^−/−^*;Stim2*^−/−^ MEF shCTR and shSec22b (*n* = 5/5 biologically independent samples; see also Supplementary Fig. S3a, b). **f** Quantification of periphagosomal calcium hotspots (arrows) and **g** phagocytic index in shCTR and shSec22b phagocytic WT MEFs loaded with Fluo-8 (blue-green ratio pseudocolor, representing fluorescence over mean cytosolic fluorescence F/F_ave_) in cells exposed to IgG-RBC (magenta) for 30 min (*n* = 7/8 biologically independent samples; 589/847 phagosomes; 256/366 cells shCTR/shSec22b). White bar = 3 µm. Bar graphs show means +SEM. White bars = 3 µm. Bar graphs show means +SEM. Error bars are omitted from traces in (**a**–**c**) for clarity.

Next, whether the Sec22-dependent changes in STIM1 recruitment impact global Ca^2+^ influx was assessed. To this end, a classical assay for assessing SOCE^22,25,26^ was employed where cells loaded with the ratiometric Ca^2+^-sensitive dye Fura-2 are exposed to Tg in Ca^2+^-free medium as above, and the maximum slope and peak amplitude of the Fura-2 Ca^2+^ signal are measured upon the re-addition of extracellular Ca^2+^. Surprisingly, despite the effect on STIM1 recruitment observed by TIRF, SOCE was unchanged in WT cells either overexpressing mCh-Sec22b in combination with YFP-STIM1 or expressing EGFP-Sec22b alone, as compared to RFP or GFP-KDEL controls, respectively (Fig. 4a, b). This was in contrast to overexpression of the Sec22b-P33 mutant, a construct where insertion of a 33-amino-acid proline tract extends the MCS gap distance, compromising Sec22b’s MCS function without affecting secretory transport^15^. In this case, global signals were faster and larger (Fig. 4b), indicating that MCS tethering by wild-type Sec22b may have both activating and inhibitory effects on the SOCE machinery that nullify one another. In support of this hypothesis, a small but significant increase in the peak amplitude was observed upon Sec22b knockdown (Fig. 4c). This effect was not due to a compensatory increase in STIM1 expression upon Sec22b knockdown, as STIM1 levels were unchanged in shSec22b MEF WT cells as compared to shCTR (Fig. 4d, Supplementary Fig. S3b). Nevertheless, this effect was still dependent on STIM1 since it was abolished in *Stim1*^−/−^ cells, which retain measurable residual influx (Fig. 4c) when these cells were transfected with shCTR or shSec22b (Fig. 4e, Supplementary Fig. S3b).

Finally, whether Sec22b could impact localized Ca^2+^ signaling during phagocytosis was assessed. To this end, an established Ca^2+^ hotspot assay based on the Ca^2+^-sensitive dye Fluo-8 was employed^5^. Phagocytic shCTR or shSec22b Fluo-8-loaded WT MEFs were exposed to IgG-coated targets, and the frequency of periphagosomal Ca^2+^ hotspots within a 750 nm distance from the phagosomal border was quantified after 30 min of ingestion. However, hotspot frequency was unchanged (Fig. 4f), and the phagocytic index was similar under these conditions (Fig. 4g). This indicates that Sec22b-dependent MCS may not be geared toward Ca^2+^ signaling, though perhaps increased Ca^2+^ influx at remaining MCS could also explain these results. Taken together, these results suggest a complex and likely indirect relationship between Sec22b and Ca^2+^ signaling. Sec22b is not required for global or localized Ca^2+^ signaling, but rather, depending on the context, it can have both positive and negative modulatory roles that may be secondary, i.e., through its influence on the shape and composition of MCS. These observations nevertheless further strengthen the idea that Sec22b localizes to phagosomal MCS and warrant further investigation on how this tethering role could impact phagocytic function.

### Sec22b-mediated MCS tethering regulates phagosomal phospholipids

**Fig. 5:**
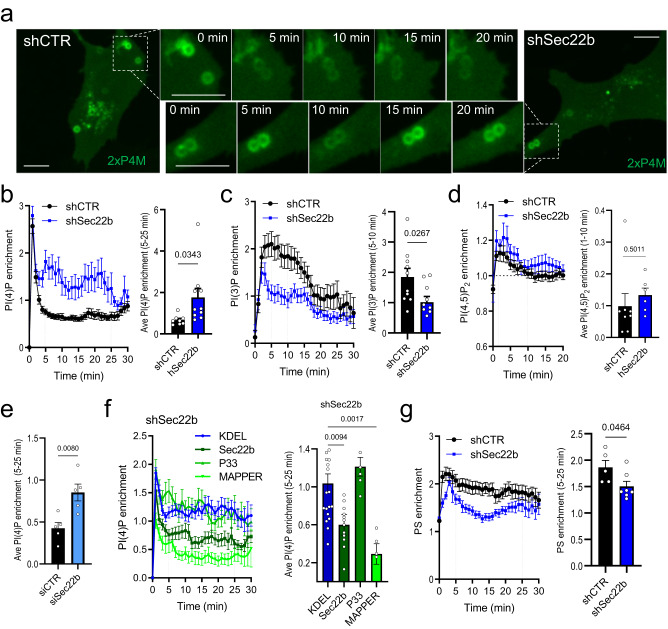
Sec22b controls the levels of phagosomal phospholipids. **a**–**c** Live spinning-disk confocal microscopy of phagosomal PI(4)P and PI(3)P in shCTR and shSec22b phagocytic MEFs transfected with GFP-2xP4M (green in (**a**), quantification in (**b**)) and TagRFP-FYVE(EE1A) (quantification in (**c**)), exposed to IgG-beads for 30–40 min (*n* = 8/10 biologically independent samples; 38/36 phagosomes; shCTR/shSec22b). **d** A similar analysis in cells expressing PI(4,5)P2 probe GFP-C1-PLCdelta-PH (*n* = 8/6 biologically independent samples; 21/9 phagosomes shCTR/shSec22b). **e** Quantification of phagosomal PI(4)P in WT phagocytic MEFs expressing GFP-2xP4M and transfected with siCTR or siSec22b (50 nM) (*n* = 5/5 biologically independent samples; 15/13 phagosomes siCTR/siSec22b; see also Supplementary Fig. S3a). **f** Quantification of phagosomal PI(4)P in shSec22b phagocytic MEFs transfected with mCh-2xP4M and either GFP-KDEL, shR-EGFP-Sec22b, EGFP-MAPPER or shR-EGFP-Sec22b-P33 (*n* = 19/14/5/6 biologically independent samples; 100/64/33/24 phagosomes KDEL/Sec22b/ MAPPER/P33; see also Supplementary Fig. S3e). **g** Quantification of phagosomal PS in shCTR and shSec22b phagocytic MEFs transfected with mRFP-Lact-C2 (*n* = 5/7 biologically independent samples; 34/52 phagosomes shCTR/shSec22b). White bars = 10 µm. Traces are means ± SEM, and bar graphs are means + SEM.

MCS are recognized as platforms for non-vesicular lipid transfer^8^. The yeast homolog Sec22 interacts with non-vesicular lipid transfer proteins (LTPs) Osh2 and Osh3, and temperature-sensitive mutations of Sec22 led to an abnormal accumulation of PM levels of phosphatidylinositol-4-phosphate (PI(4)P)^15^. Thus, since Sec22b-dependent MCS were not majorly involved in Ca^2+^ signaling, they might be instead involved in regulating phagosomal PI(4)P levels by promoting non-vesicular lipid transfer at phagosomal MCS. To test this hypothesis, we utilized the PI(4)P probe 2xP4M^27^ previously employed to investigate phagosomal PI(4)P levels^28^. Phagocytic shCTR and shSec22b MEFs were co-transfected with 2xP4M-GFP and the phosphatidylinositol-3-phosphate (PI(3)P) probe tagRFP-FYVE^29^, intended to serve as control. Cells were exposed to IgG-coupled beads, and live confocal images were recorded for 30–40 min to follow phagosomal lipid enrichment over time. In shCTR cells, a strong initial peak followed by a rapid decrease of phagosomal PI(4)P was observed, after which two populations were apparent. In the majority, phagosomal PI(4)P remained low, and in the minority, phagosomes displayed some continuing fluctuations in PI(4)P, similar to macrophages^28^ (Fig. 5a). In shSec22b cells, whereas the initial peak increase in PI(4)P was comparable to controls, the fraction of phagosomes with continuing PI(4)P fluctuation was increased such that the average phagosomal PI(4)P was ⁓2.5-fold higher 5–25 min after the initial peak (Fig. 5a, b). In addition, PI(3)P phagosomal levels were ⁓45% lower in shSec22b cells 5–10 min after ingestion (Fig. 5c). However, this phenotype did not extend to all phagosomal phosphoinositides as PI(4,5)P2 levels, measured using the PLCδ-PH domain probe^30^, were similar to controls (Fig. 5d). Sec22b was also transiently depleted, by ⁓63% (Supplementary Fig. S4a–d), using an siRNA pool (siSec22b) containing sequences that were distinct from the shSec22b sequence. Similarly, phagosomal PI(4)P was ⁓2-fold higher in siSec22b as compared to siCTR in phagocytic MEF cells expressing 2xP4M-GFP without co-expression of any other probe (Fig. 5e). These results argue against confounding off-target effects of the shRNA sequence or the co-expression of the PI(3)P probe. Importantly, re-expression of an shRNA-resistant (shR) EGFP-tagged wild-type Sec22b as compared to GFP-KDEL in combination with 2xP4M-mCherry rescued the PI(4)P phenotype, whereas shR-Sec22b-P33 did not (Fig. 5f), despite robust phagosomal recruitment (Supplementary Fig. S4e). Moreover, increasing MCS tethering by overexpression of MAPPER also leads to the rescue of phagosomal PI(4)P levels in Sec22b-depleted cells (Fig. 5f). At the PM and Golgi PI(4)P levels are regulated by LTPs in a counter-exchange mechanism with phosphatidylserine (PS) or cholesterol, respectively, that is powered by the dephosphorylation of PI(4)P in the ER by Sac1^31^. Thus, whether PS is affected by Sec22b depletion was examined by overexpressing the RFP-tagged PS-binding probe Lact-C2^32^. Indeed, in Sec22b-depleted cells, a ⁓20% decrease in phagosomal PS levels was detected (Fig. 5g). Together, these data are consistent with a putative bidirectional transfer of PI(4)P and PS across ER-Phg MCS.

### ORP8 contributes to phagosomal lipid regulation by Sec22b

**Fig. 6:**
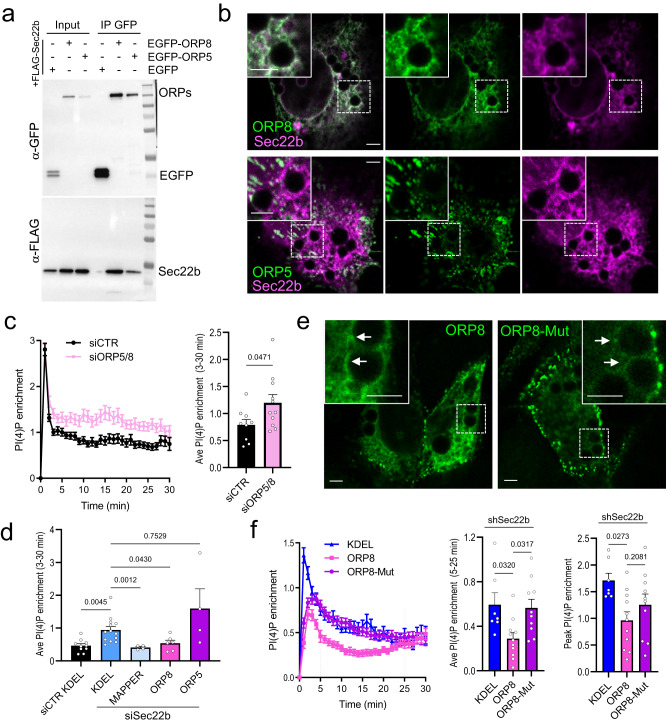
Sec22b interaction with ORP8 regulates phagosomal PI(4)P. **a** Western blot of whole-cell lysates (input) and GFP-trap agarose beads immunoprecipitates (IP) of HeLa cells transfected with FLAG-Sec22b and either EGFP-ORP8, EGFP-ORP5 or EGFP control, using anti-GFP and anti-FLAG. **b** Phagocytic MEFs co-transfected with mCh-Sec22b (magenta) and either EGFP-ORP8 (green, top panels) or EGFP-ORP5 (green, bottom panels). **c** Quantification of phagosomal PI(4)P in WT MEFs transfected with GFP-2xP4M and either siCTR (100 nM) or siORP5/8 (50 nM each); *n* = 9/11 biologically independent samples, 72/79 phagosomes in siCTR/siORP5/8; see also Supplementary Fig. S3b–d. **d** Quantification of phagosomal PI(4)P in WT phagocytic MEFs transfected with mCh-2xP4M and siCTR+GFP-KDEL, or siSec22b+GFP-KDEL, +EGFP-MAPPER, +EGFP-ORP8 or +EGFP-ORP5. (*n* = 10, 13/4/6/4 biologically independent samples, 61, 81/29/28/26 phagosomes siCTR+KDEL, siSec22b+KDEL/ MAPPER/ ORP8/ ORP5). **e** Phagocytic shSec22b MEFs transfected with EGFP-ORP8 or EGFP-ORP8-H514A-H515A (ORP8-Mut). **f** Quantification of initial peak levels as well as phagosomal PI(4)P in shSec22b phagocytic MEFs transfected with mCh-2xP4M and GFP-KDEL, EGFP-ORP8 or EGFP-ORP8-Mut. (*n* = 7/11/10 biologically independent samples, 111/123/185 phagosomes KDEL/ORP8/ORP8-Mut). IgG beads were added for 30 min. White bars = 3 µm. Bar graphs are means +SEM.

Sec22 was reported to interact with LTPs Osh2 and Osh3 in yeast^15^, which are homologs of the mammalian ORP family of LTPs^31,33^. In mammals, the closely related ORP5 (also called Osbpl5) and ORP8 isoforms mediate lipid exchange of PI(4)P at the PM as well as at endo-lysosomal compartments^31,34^. Thus, we asked whether ORP5 or 8 could contribute to Sec22b-regulated lipid exchange at phagosomes. After unfruitful attempts to immunoprecipitate endogenous or overexpressed Sec22b in MEFs, FLAG-Sec22b was co-expressed with EGFP-tagged ORP5 or ORP8 or with cytosolic EGFP as control in HeLa cells where transfection efficiency is higher (Fig. 6a). Sec22b co-immunoprecipitated with both ORP5 and ORP8, but not with cytosolic EGFP, suggesting that interactions between Sec22b and LTPs are conserved in mammalian cells. In addition, phagocytic MEFs were co-transfected with either EGFP-ORP5 or EGFP-ORP8 and mCh-Sec22b. ORP5 overlapped with Sec22b in peripheral patches reminiscent of ER-PM junctions^34^ but overlap at periphagosomal locations was not salient (Fig. 6b). In contrast, co-expression of mCh-Sec22b and EGFP-ORP8 showed a striking overlap in both the ER and around phagosomes (Fig. 6b). We next investigated if ORP5/8 are involved in transporting lipids across ER-Phg MCS using siRNA. Although the siRNA pools employed were inefficient at downregulating ORP5 and 8 protein expression individually, when applied simultaneously, a downregulation of 59% and 61% for ORP5 and 8, respectively, was observed (Supplementary Fig. S4b–d). The phagosomal PI(4)P dynamics were then assessed in ORP5/8 depleted cells as above. ORP5/8 depletion resulted in a ⁓1.5-fold increase in phagosomal PI(4)P levels, phenocopying the knockdown of Sec22b (Fig. 6c). Moreover, overexpression of EGFP-ORP8, but not EGFP-ORP5, rescued the phenotype in Sec22b-depleted cells, similar to MAPPER (Fig. 6d). To test whether the lipid transfer function was specifically required for the effect of ORP8 on phagosomal PI(4)P, a mutant EGFP-ORP8 harboring two point mutations H514A-H515A (ORP8-Mut), which abrogates lipid transfer but not tethering^34^ was employed. EGFP-ORP8-Mut appeared to be more robustly recruited to PM-MCS and less reticular than the wild-type protein, and recruitment to phagosomes was less prominent (Fig. 6e). ORP8 but not ORP8-Mut reduced phagosomal PI(4)P by ⁓35% at peak and by ⁓48% at 5–25 min after ingestion (Fig. 6f), indicating the lipid transfer function of ORP8 is required for its ability to localize to phagosomes and to rescue the Sec22b knockdown phenotype. These data indicate that the loss of Sec22b can be compensated by overexpressing ORP8 and that ORP8 does not require Sec22b to be recruited to MCS, although we cannot exclude that such recruitment may be more efficient in the presence of Sec22b. Taken together, our data point toward a function of Sec22b in the formation or stabilization of ER-Phg MCS and the recruitment of ORP8, which transports PI(4)P from the phagosome to the ER and PS from the ER to phagosomes.

### Sec22b-mediated tethering controls phagosomal maturation

**Fig. 7:**
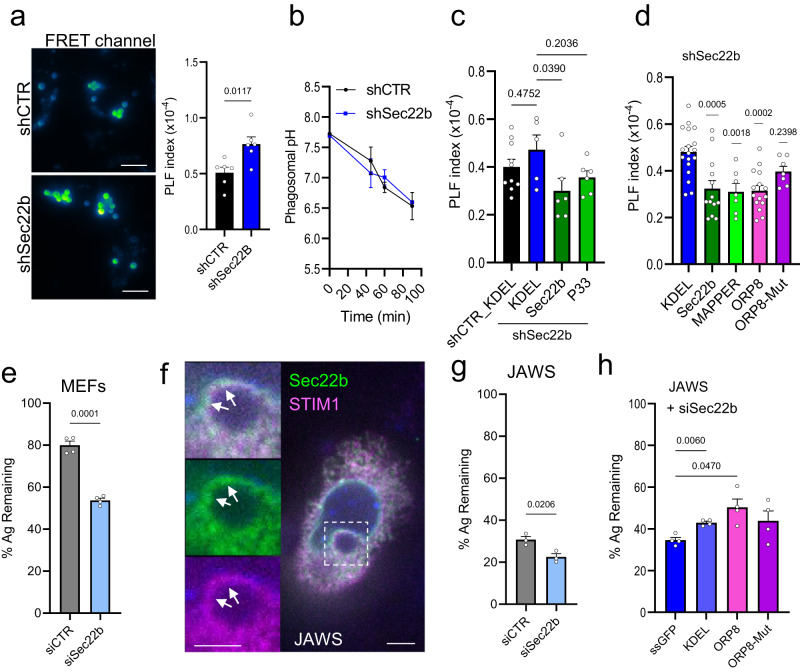
Sec22b MCS tethering regulates phagosomal maturation. **a** Phagolysosome fusion analysis (PLF index) in phagocytic shCTR and shSec22b MEFs loaded with FRET acceptor AF594-HA and exposed to FRET donor-coupled AF488-IgG-beads for 90 min. **a** Images show representative blue-green pseudocolor of the maximum projection of FRET signal stacks (480/630 ex/em, 15 planes/0.8 µm spacing) in shCTR and shSec22b (*n* = 6/6 biologically independent samples, 1071/1060 phagosomes shCTR/shSec22b; see also Supplementary Fig. S5a, bar = 10 µm). **b** Phagosomal pH in shCTR and shSec22b phagocyotic MEFs 45, 60 and 90 min after exposure of cells to IgG-FITC-RBC. (*n* = 6/6 biologically independent samples, 4163/3049 phagosomes) **c** PLF index in phagocytic shCTR and shSec22b MEFs transfected with GFP-KDEL, shR-EGFP-Sec22b or shR-EGFP-Sec22b-P33. (*n* = 10;5/6/6 biologically independent samples, 4757;1363/1426/974 phagosomes for shCTR+KDEL; shSec22b+KDEL /+Sec22b/+P33. See also Supplementary Fig. S5b–d). **d** PLF index in phagocytic shSec22b MEFs transfected with GFP-KDEL, shR-EGFP-Sec22b, EGFP-MAPPER, EGFP-ORP8 or EGFP-ORP8-Mut. (*n* = 11/6/15/7/7 biologically independent samples, 4311/2896/476/2080/566 phagosomes KDEL /Sec22b/ MAPPER/ ORP8/ ORP8-Mut. See also Supplementary Fig. S5e). **e** Quantification of mean phagosomal antigen intensity (anti-EPEA) expressed as a percentage of the mean intensity of non-ingested beads (% Ag remaining) after 90 min incubation of siCTR and siSec22b (50 nM) transfected phagocytic MEFs with EPEA-peptide coupled, IgG opsonized beads. (*n* = 4/4 biologically independent samples, 81/99 phagosomes siCTR/siSec22b). **f** JAWS DCs transfected with EGFP-Sec22b and mCh-STIM1. Arrows: periphagosomal puncta containing Sec22b and STIM1. Bar = 3 µm. **g** Quantification of phagosomal antigen degradation as in (**e**) in JAWS cells transfected with siCTR or siSec22b (50 nM) (*n* = 4/4 biologically independent samples, 727/958 phagosomes siCTR/siSec22b. See also Supplementary Fig. S5f–g) **h** Quantification of phagosomal antigen degradation as in (**e**) in JAWS cells co-transfected with siSec22b and soluble secreted GFP (ssGFP), GFP-KDEL, EGFP-ORP8 or EGFP-ORP8-mut. (*n* = 4/4/4/4 biologically independent samples, 753/625/680/1034 phagosomes in siSec22b+ ssGFP/GFP-KDEL/EGFP-ORP8/EGFP-ORP8-mut; see also Supplementary Fig. S5h). Graphs show means + SEM.

We next investigated the physiological relevance of this lipid transfer mechanism at ER-Phg MCS. PI(4)P promotes phagosome maturation and phagolysosome fusion by recruiting Rab7 effectors^28,35^. As discussed above, increased phagosomal LAMP1 and an exacerbated degradation of ingested antigens were observed upon Sec22b knockdown in DCs, suggesting an increase in phagolysosome fusion, although no mechanism was provided^18^. We thus employed an established Förster resonant energy transfer (FRET)-based phagolysosome fusion assay^7^ to examine this process in our model system. Phagocytic MEFs were loaded with the impermeant FRET acceptor Alexa Fluor (AF) 594-HA, which accumulated in lysosomes. Cells were then exposed to IgG-beads coupled to the FRET donor AF488, and the FRET, total acceptor and total donor signals were measured by microscopy. After 90 min of phagocytosis, phagolysosome fusion (PLF index) was increased by ⁓1.5-fold in Sec22b-depleted cells as compared to controls (Fig. 7a), whereas the rate of phagocytosis (phagocytic index) and total lysosomal loading was similar between the two conditions (Supplementary Fig. S5a). Phagosomal pH was also similar upon Sec22b knockdown (Fig. 7b), which is consistent with results observed in DCs^18^, with previous reports suggesting that V-ATPase delivery to phagosomes precedes phagolysosome fusion^36,37^, as well as our own observations that when phagolysosome fusion is decreased in *Stim1*^−/−^ cells, pH remains unchanged^7^. We then determined whether the PLF index could be rescued by overexpression of Sec22b or different MCS tethering and control constructs to verify whether the effects observed on PI(4)P mirrored effects on phagolysosome fusion. The first observation was that expression of GFP-KDEL alone appeared to reduce the PLF index in general (compare Fig. 7a with Fig. 7c) with a greater effect in shSec22b cells, leading to a diminished dynamic range and loss of a significant difference between the shCTR+KDEL and shSec22b+KDEL conditions (Fig. 7c). On the other hand, overexpression of shR-Sec22b lowered the PLF index compared to KDEL in shSec22b cells at both low and high phagocytic rates by 35 and 43%, respectively (Fig. 7d and Supplementary Fig. S5b, c). Although GFP signals represented at most 10% of the much brighter donor dye (AF488) channel signal and were mostly excluded by a restrictive phagosomal segmentation, one caveat of this technique is that differences in construct expression levels cannot be entirely excluded from the PLF index calculation. While most EGFP constructs appeared to be expressed at similar levels, GFP-KDEL fluorescence was visibly lower than EGFP-Sec22b, and thus we also calculated a FRET ratio independently of the green channel fluorescence (FRET signal normalized to total acceptor dye loading). In this case, we still observed a 27% decrease in the FRET ratio upon expression of Sec22b (Supplementary Fig. S5b), supporting the idea that Sec22b overexpression indeed directly lowers phagolysosome fusion. In contrast, although a trend toward a partial effect could be discerned, the PLF index upon overexpression of shR-Sec22b-P33, in the context of similar phagocytic index and total acceptor loading, was not significantly different from that of KDEL (Fig. 7c, Supplementary Fig. S5d). Moreover, both MAPPER and ORP8 but not ORP8-Mut lowered the PLF index by ⁓35% without affecting phagocytic rates (Fig. 7d, Supplementary Fig. S5e). However, in the case of MAPPER and ORP8-Mut, the total lysosomal loading of the acceptor dye was 30% higher than with KDEL, suggesting more generalized effects on the endocytic pathway may contribute to this phenotype. However, since the PLF index is normalized to total loading and since MAPPER and ORP8-Mut had opposite effects on the PLF index while having similar effects on total acceptor loading, differences in the latter cannot fully explain the changes in PLF observed. As an alternative approach, to both validate these findings using siRNA as well as to confirm an impact on phagosomal maturation, we determined whether siSec22b transfection would impact antigen degradation. To this end, phagocytic MEFs transfected with siCTR or siSec22b were exposed to beads coupled to peptides bearing the small tag EPEA^38^ for 90 min. The bead-associated fluorescence, normalized to non-ingested beads (percent antigen remaining on beads), was quantified in fixed cells immunostained with anti-EPEA. Indeed, siRNA-mediated knockdown also led to exacerbated antigen degradation in MEF cells (Fig. 7e).

Finally, to verify that the results obtained in MEFs are generalizable to other phagocytes, we made use of the DC cell line JAWSII (JAWS), where Sec22b-dependent differences in phagosomal maturation have been previously observed^18^. First, we verified that Sec22b and STIM1, serving as an MCS marker, also co-localize in periphagosomal puncta (Fig. 7f). In addition, siRNA mediated Sec22b knockdown (50% efficiency, Supplementary Fig. S5f) also led to a small but significant exacerbation of phagosomal antigen degradation in JAWS DCs (Fig. 7g) without impacting phagocytic rates (Supplementary Fig. S5g). Then, to determine whether ORP8-mediated lipid transfer could influence phagosomal maturation in DCs, we performed the same assay in JAWS DCs co-transfected with siSec22b and ER-targeted GFP controls or ORP8 wild-type and mutant constructs. Since we noticed that in JAWS GFP-KDEL transfection displayed some toxicity (fewer cells after transfection compared to all other constructs), we also included as a control a secreted soluble GFP (ssGFP) that retains the same ER-import signal peptide but lacks the KDEL ER-retention signal^39^. Interestingly, the phagosomal antigen degradation phenotype induced by Sec22b downregulation was reversed by co-expression of ORP8 but not ORP8-Mut when compared to ssGFP (Fig. 7h) without affecting phagocytic rate (Supplementary Fig. S5h). Thus, while other mechanisms likely contribute, together, these data suggest that Sec22b regulates phagosome maturation in MEFs and DCs, at least in part, by recruiting ORP8 to phagosomal MCS.

## Discussion

In our previous studies in neutrophils and DCs, STIM1-dependent ER-phagosome MCS were strongly associated with localized Ca^2+^ hotspots, yet residual hotspots and contacts persisted in the absence of STIM proteins, leading us to search for other MCS tethers^5,6^. Indeed, comparing EM quantification of phagosomal MCS in our two studies revealed that DC phagosomes have a 5-fold higher frequency of MCS as compared to neutrophils^5,7^, suggesting that contacts may be particularly important for DCs. A potential connection between Sec22b and STIM1 was hinted at by the study of Galli and colleagues in HeLa cells, where luminal ER Ca^2+^ refilling was reduced and delayed upon Sec22b-P33 expression, although Sec22b knockdown did not impact global Ca^2+^ signals^15^. In the present study, we employed phagocytic MEFs as a genetically tractable cell model that resemble DCs in that phagosomal maturation is much milder than macrophages^6,40^. In MEFs, Sec22b knockdown as well as P33 overexpression both increased global cytosolic Ca^2+^ signals (Fig. 4b, c), whereas overexpression of Sec22b had a dramatic effect on STIM1 recruitment to plasma membrane MCS. Yet surprisingly, this did not translate to changes in cytosolic signals. Conceivably, Sec22b stabilizes MCS allowing a faster infiltration of STIM molecules, yet its presence may interfere with STIM-ORAI pairing either by recruiting components that displace Ca^2+^ channels, by changing the local lipid environment, or by regulating MCS gap distance—parameters shown to modulate Ca^2+^ channel recruitment and activity^41–43^. Such parameters may be cell type-dependent, explaining differences from previous reports. Sec22b downregulation did not change the frequency of local signals (Fig. 4f), which are known to drive lysosome fusion^2,44^. Since the low levels of BAPTA required to visualize hotspots precludes estimation of Ca^2+^ concentrations, an undetected increase in the size (rather than frequency) of Ca^2+^ hotspots, in addition to global Ca^2+^ effects, could contribute to increased phagolysosome fusion upon Sec22b depletion. However, the dramatic effect of Sec22b depletion on PI(4)P we now show (Fig. 5) is perhaps more likely to play a greater role since PI(4)P has been shown to drive phagolysosome fusion^28,35^. On the other hand, inhibition of ER Ca^2+^ release because of reduced refilling^15^ could explain why Sec22b-P33 still shows a tendency toward reduced phagolysosome fusion (Fig. 7c), despite its complete inability to rescue levels of PI(4)P (Fig. 5f). Regardless, it should be kept in mind that Sec22b may have subtle effects on Ca^2+^ signaling which may be more or less important depending on cell type.

Several studies detect Sec22b recruitment to macrophage and DC phagosomes by immunofluorescence, Western blot or proteomic analyses of isolated phagosomes^17,18,45–47^, where in macrophages, its downregulation led to increased phagocytic rates^17^. In DCs, Sec22b knockdown did not impact the phagocytic rate but induced an unexplained yet clear increase in LAMP1 recruitment, cathepsin activity and phagosomal antigen degradation^18^. Interestingly, in a recent study, most but not all of the Sec22b present on isolated DC phagosomes was shed by proteinase K treatment^47^. The present study, showing that in addition to ERGIC-localized Sec22b, ER-localized Sec22b associates with phagosomes through MCS, now provides an explanation for these results. Here, increased phagolysosome fusion was not due to off-target effects, as has been suggested for the effect of shSec22b on cross-presentation^48^, since an siRNA pool comprised of sequences distinct from this shRNA produced the same result, and since Sec22b re-expression rescued the phenotype (Fig. 7c, d). Our initial hypothesis was that changes in Ca^2+^ might drive the effect^2,49^. Instead, our data pointed to changes in phagosomal phospholipids through the recruitment of LTPs at phagosomal MCS. In Sec22b knockdown cells, reducing phagosomal PI(4)P through MAPPER and ORP8 expression, both of which only minorly impact cytosolic Ca^2+^^24,50^, correlated well with changes in phagolysosome fusion. This is logical since PI(4)P is a docking site for Rab7 effector RILP, thus promoting lysosomal fusion^28,35^. However, we also detected decreases in phagosomal PS and PI(3)P, which may contribute to changes in phagosomal maturation. Whereas phagosomal PS regulates surface charge and recruitment of c-Src, PI(3)P recruits effectors such as VPS34 and PI(3,5)P2-kinase PikFYVE promoting phagolysosome fusion^32,51,52^. Their decrease would thus be predicted rather to reduce fusion with endocytic vesicles. In contrast, PI(3)P binding to the NADPH oxidase subunit p40^*phox*^(also called NCF4) promotes phagosomal reactive oxygen species (ROS) production, which delays phagosomal maturation by preventing acidification and is critical for efficient cross-presentation^37,40,53^. However, in MEFs, ROS production is presumably negligible, and in macrophages, Sec22b knockdown did not impact Nox2-mediated ROS production^54^, although it did reduce iNOS-^47^ and IRE1α-^54^ dependent ROS at late (<8 h) timepoints in DCs and macrophages, respectively. How Sec22b regulates PI(3)P is still obscure, although feedback coordination with PI(4)P could be involved. Whether changes in PI(3)P contribute to knockdown phenotypes in DCs would be interesting to determine in future studies.

Finally, the remaining questions include: what is the impact of Sec22b-regulated phagolysosome fusion on cross-presentation, does this relate to ERGIC fusion, and if so, how? Yet answering these questions is not trivial. Reduced phagosomal proteolysis is predicted to favor cross-presentation by limiting antigenic peptide destruction^55,56^. However, cell-surface expression of co-stimulatory/co-inhibitory molecules and cytokine secretion, which contribute profoundly to T cell responses, may also be affected by changes in lysosome behavior. Indeed, Sec22b regulation of the nuclear translocation of NF-kB, which controls cell-surface expression of many co-stimulatory molecules, as well as IL-6, TNFα and IL-1β secretion, were only recently discovered, the latter via secretory autophagy^47,57^. Since autophagy was also recently linked to levels of MHC-I^58^, determination of the final effect on cross-presentation will require untangling the lysosomal impact on all of these factors, and our current research efforts are centered on these questions.

Importantly, the impact of Sec22b on cross-presentation has, up to now, always been assumed to rely on its ability to deliver ER proteins to phagosomes through the fusion of ERGIC vesicles^18,55,56,59,60^. MCS are non-fusogenic, stable associations between organelles that survive biochemical fractionation^8^. Our observations that Sec22b resides in and regulates phagosomal MCS, therefore, now beg a re-interpretation of previous studies investigating the Sec22b-mediated recruitment of ER proteins to phagosomes since a portion of the ER proteins detected are likely still contained within MCS. Indeed, this explains the punctate rather than continuous immunostaining of antigen-loaded MHC-I molecules around phagosomes^61^, the presence of empty (lacking a peptide) or endoglycosidase H-sensitive MHC-I molecules on isolated phagosomes^55,62^, and the major loss of Sec22b on isolated phagosomes after 1 h proteinase K digestion^47^. Our results do not rule out a contribution of ERGIC fusion to phagosomal maturation, and indeed we observe a close association between phagosomes and structures containing Sec22b and ERGIC markers such as Stx5 and ERGIC-53 even in MEFs (Figs. 1 and 2, Supplementary Figs. S1 and S2), as has been previously reported in DCs^18,47,60^. Nevertheless, a shift in the balance of Sec22b-mediated MCS formation versus ERGIC fusion, caused, for example, by changes in basal levels of lipid loading, autophagy, ER stress or post-translational modifications of Sec22b, may be confounding and could help explain discrepancies between prior studies. Future studies that distinguish between effects on MCS and fusogenic pathways will surely help formulate a more accurate picture of the complex biology of this important trafficking regulator.

## Methods

### Reagents

The following antibodies (antibody name/catalog#/dilution, IF: immunofluorescence, WB: Western blot) were purchased from: Synaptic Systems (SYSY): rabbit anti-Sec22b (186003/1:200 IF, 1:1000 WB), rabbit anti-Stx5 (110053/1:100 IF, 1:1000 WB); Santa Cruz: mouse anti-Sec22b (29-F7) (sc-101267/1:100); Cell Signaling: mouse anti-c-myc antibody (9B11) (2276/1:100), rabbit anti-STIM2 (4917S/1:1000); Thermo: mouse anti-CD16-CD32 (Fc-Block, 14-0161-85, 1:200), rabbit anti-ORP5 (PA5-18221/1:500), goat-anti-rabbit Alexa Fluor 555 (A21428/1:1000), goat-anti-mouse Alexa Fluor 647 (A21235/1:1000), goat-anti-human Alexa Fluor 633 (A-21091/1:500); GeneTex: rabbit anti-ORP8 (GTX121273/1:500); Sigma: mouse anti-α-tubulin (T9026/1:5000), rabbit anti-sheep red blood cell (sRBC, S1389/1:40), mouse anti-FLAG-M2 (F1804/1:1000); mouse anti-GFP (11814460001/1:1000); BD Biosciences: mouse anti-GOK/STIM1 (610954/1:100); ABCD Antibodies: human IgG1-anti-EPEA (AI215-H1/1:100); Bio-Rad: goat anti-rabbit IgG (H+L) HRP conjugate (170-6515/1:10000), goat anti-mouse IgG (H+L) HRP conjugate (170-6516/1:10000), Innovative Research: human IgG protein A purified (hIgG) (IR-HU-GF); Jackson ImmunoResearch: donkey-anti-mouse Alexa Fluor 488 (715-545-150/1:800), goat-anti-rabbit Alexa Fluor 647 (111-605-003/1:1000), goat-anti-rabbit DyLight 405 (111-475-003/1:800). Interfering RNA was purchased from: Santa Cruz: mouse siSec22b (sc-153306, siRNA pool/50 nM), siCTR (sc-37007/50-100 nM); Dharmacon/GE Healthcare: ON-TARGETplus siRNA mouse siORP8 (Osbpl8 (237542) SMARTpool SO-2755574G/50 nM), ON-TARGETplus siRNA mouse siORP5 (Osbpl5 (79169), SMARTpool so-2791415G/50 nM); ON-TARGETplus siRNA mouse siStx5a (L-063346-01/10 nM) mouse shSec22b lentiviral clone (TRC Clone ID: TRCN0000115089); Sigma: shCTR non-target control particles SCH002V Mission shRNA Lentiviral clone. All lentiviral particles were produced in Lenti-X 293T cells using the Lenti-X HTS Packaging System (Takara, Japan) according to the manufacturer’s instructions. Lentiviral titers were determined using the LentiX-p24 Rapid Titer ELISA kit (Takara). All cell culture reagents were obtained from ThermoFisher Scientific, and all chemicals were purchased from Sigma-Aldrich unless otherwise stated.

### Plasmids

The following plasmids were a gift from the laboratory of Prof. Nicolas Demaurex (University of Geneva): pcDNA3-myc-FCGR2A (Fc receptor)^51^, pEGFP-FCGR2A^51^ p-DS-XB-GFP-MAPPER-Long^24^, pCMV-ER-GFP (GFP-KDEL) (Thermo), pAmaxa-ER-TagRFP (RFP-KDEL)^63^, YFP-STIM1 (Addgene plasmid # 18857)^64^, GFP-C1-PLCdelta-PH (Addgene plasmid # 21179)^30^. Soluble secreted GFP (ssGFP) was a gift from Drs. Richard Bouley and Dennis Brown^39^. Plasmids pEGFP-ORP5, pEGFP-ORP8 and pFLAG-Sec22b were generated in the Giordano laboratory^65^. GFP-ERGIC-53 was a gift from Drs. Houchaima Ben Tekaya and Dr. Jean Gruenberg (University of Geneva)^66^. pCMV-EGFP-Sec22b, pCMV-EGFP-Sec22b-P33, pCMV-mCherry-Sec22b were gifts from Drs. Thierry Galli and Christian Vannier (INSERM, Paris)^15^. mRFP-Lact-C2 (Addgene plasmid # 74061)^32^, GFP- and mCherry-2xP4M^27^ were kind gifts from Dr. Sergio Grinstein (University of Toronto). pRS424GFP-FYVE(EEA1) was a gift from Scott Emr (Addgene plasmid # 36096). TagRFP-FYVE(EE1A) was subcloned from pRS424GFP-FYVE(EEA1) into pTagRFP-C (Evrogen) and pEGFP-C1 (Clontech) using EcoRI/KpnI restriction enzymes (NEB). EGFP-Sec22b-shR (Addgene deposit ID:208358) and EGFP-Sec22b-P33-shR (Addgene deposit ID:208359) were generated by site-directed mutagenesis (F: CTTCTGAATGAAGGTGTCGAACTCGATAAAAGAATAAGGCCTAGACACAGTGGGC; R: GCCCACTGTGTCTAGGCCTTATTCTTTTATCGAGTTCGACACCTTCATTCAGAAG) using the Q5 Site-Directed Mutagenesis Kit (NEB). pEGFP-ORP8-H514A-H515A (ORP8-Mut Addgene deposit ID: 208360) was similarly generated (F: ACAGGTGTCCgctgctCCACCAATATCTG, R: TCAGCAATATAAAAAGTTTTGC).

### Cell culture, transfection and transduction

Wild-type mouse embryonic fibroblasts (MEF, ATCC CRL-2991), *Stim1*^*−/−*^ MEFs^67^, *Stim1*^*−/−*^*;Stim2*^−/−^ MEFs^68^, and HeLa cells (ECACC, 93021013, a gift from N. Demaurex) were grown in DMEM (22320) containing 10% fetal calf serum, 1% penicillin/streptomycin (pen/strep), at 37 °C and 5% CO_2_ and were passaged twice a week. MEF cells were used between passages 5 and 50. Hela cells were authenticated by short tandem repeat genomic profiling (Microsynth) in the Demaurex laboratory. All cell lines were tested every 6–12 months either by PCR (LookOut kit, Sigma/MErck) or Mycostrips (Invivogen, ep-mysnc-50) and were negative. Cells were transfected using Lipofectamine 2000 in full medium without antibiotics for 4–6 h with cells at 50–60% 1–2 days after seeding for plasmids or at the same time as seeding (reverse transfection) for transfections containing siRNA. Lentivirus transduction was performed by centrifuging cells and viral particles at 5 MOI in complete medium supplemented with 8 μg/ml polybrene at 500×*g* at 37 °C for 1 h. To obtain stable cell lines, puromycin (10 µg/ml) selection was performed 2 days after transduction. Stable cell lines (shCTR, shSec22b) were maintained in a medium supplemented with (10 µg/ml) puromycin. JAWSII DCs (ATCC CRL-11904) were grown in Alpha-MEM with ribonucleosides, deoxyribonucleosides (22571) medium containing 4 mM L-glutamine (25030), 1 mM sodium pyruvate (58636), 20% fetal calf serum, 1% pen/strep, 5 ng/ml murine GM-CSF (Peprotech, 315-03), and used between passage 19 and 40. Cells were transfected using the Amaxa nucleofection kit for mouse dendritic cells (Lonza, VPA-1011) according to manufacturer’s instructions and program Y-001.

### Phagocytic target preparation

In this, 3 μm carboxyl polystyrene microspheres (Spherotech/ Cat No. CP-30-10) were covalently coupled with hIgG by washing 3 times in sterile PBS at maximum speed (18,000×*g*) at 4 °C, activating with 50 mM 1-Ethyl-3-(3-dimethylaminopropyl)carbodiimide hydrochloride(EDC-HCl) (Carl Roth/2156.1) for 15 min in PBS at room temperature (RT) with rigorous shaking, followed by 3 washes at 4 °C in 0.1 M Na_2_B_4_O_7_ buffer (pH 8.0). Then, 6 mg of hIgG was added to the beads and incubated overnight at 4 °C on a shaker. The following day, the beads were washed 2× with 250 mM glycine/PBS, followed by two washes in PBS. For fluorescent IgG-bead preparation, 20 μg/ml Alpha Fluor 488 amine (AF488-Amine) (AAT Bioquest/Cat No. 1705) was added after EDC activation, and particles were incubated with the dye for 30 min at RT with agitation followed by one wash before adding hIgG. A similar method was used to couple 3 μm amino polystyrene microspheres (Polysciences/ POL17145-5) to 0.5 mg/ml peptide custom EPEA-tagged peptide (Proteogenix, CGGMFVESIINFEKLTEWFRSNVMGGEPEA), using 2.5 mM sulfo-SMCC (MedChem Express/HY-D0975) in PBS/2 mM EDTA /5 mM TCEP as cross-linker. Gluteraldehyde-stabilized sheep RBCs were incubated for 1 h in 0.1% NaBH_4_, washed 3x in 0.1 M Na_2_CO_3_ (pH 9.3 or pH 8.0) followed by incubation with 1 mg/ml fluorescein isothiocyanate (FITC) or pHrodo Red, succinimidyl ester (Thermo/P36600), respectively, for 4 h and washed 3x in PBS. Sheep RBCs were opsonized in rabbit anti-sRBC for 1 h at 37 °C, followed by 3 washes in PBS at 4 °C just prior to use. Prepared beads and RBCs were resuspended in PBS+1% pen/strep, counted using a Countess (Thermo) cell counter, and added to cells at a 10:1 target:cell ratio unless otherwise indicated. All buffers were sterile-filtered using 0.2 µm filters.

### Immunofluorescence

MEF cells seeded on 0.17 thickness 12 mm coverslips (Carl Roth) were fixed in 4% paraformaldehyde (PFA, Electron Microscopy Sciences)/PBS for 30 min, and JAWSII cells were fixed in 2% PFA/10 min. For immunostainings, cells were permeabilized in 0.1% TritonX-100/PBS, reduced with fresh 0.1% NaBH_4_ (Carl Roth)/PBS for 10 min, treated with Image-IT-Fx (Thermo)for 30 min, blocked in 1% BSA/PBS+Fc-Block when appropriate (blocking buffer) for 30 min, and incubated overnight at 4 °C in primary antibody and for 1 h at RT in secondary antibody. Coverslips were mounted in SlowFade Gold or Prolong Diamond (Thermo). Images were acquired either using a Nikon A1r confocal microscope system/60×1.4 CFI Plan Apochromat objective or a Zeiss LSM700 system/Plan-Apochromat 63x /1.4 objective. Z-stacks were taken at 0.5 µm intervals. Quantification of MAPPER recruitment to phagosomes or EPEA-tag immunofluorescence was performed using ImageJ. For MAPPER, A 0.2 µm (~4 pixels) ring beyond the phagosomal border (based on the brightfield image) was examined for regions above threshold (manually defined per cell). The number of MAPPER punctate structures (>0.01 µm^2^/4 pixel area) around each phagosome is reported. For EPEA circle regions of interest were drawn using brightfield images to select beads and EPEA fluorescence quantified from sum projections. Percent antigen remaining is defined as the fluorescence of cell-associated beads divided by the fluorescence of beads not associated with cells.

### Electron microscopy

Transmission electron microscopy (TEM) was performed at the Pôle Facultaire de Microscopie Electronique at the University of Geneva. Cells were fixed in 2% glutaraldehyde/0.1 M sodium phosphate buffer, pH 7.4. Cell pellets were washed 1x in 0.1 M phosphate buffer prior to dehydration in ethanol and embedded in Epon (Electron Microscopy Sciences). En bloc staining was performed with Reynold’s (lead citrate, Electron Microscopy Sciences), postfixation with osmium tetroxide. Then, 20 nm sections were imaged on a Tecnai transmission electron microscope (FEI), and quantification was performed manually using ImageJ. MCS were defined as electron-dense membranes resembling flat cisternae within 30 nm of the phagosomal membrane. Only phagosome slices containing a cross-section of more than 1 μm in diameter were considered.

### Correlation light electron microscopy (CLEM)

CLEM was performed according to protocols established in prior studies as follows^7,69^. Transfected cells seeded on Grid-500 polymer dishes (Ibidi) and exposed to phagocytic targets were fixed in 4% PFA for 30 min prior to high-resolution confocal and brightfield imaging. After light image acquisition dish was re-fixed in 2.5% glutaraldehyde/2% PFA/2 mM CaCl_2_/0.15 M sodium cacodylate buffer (pH 7.4) for 3 h, dehydrated and embedded in Epon and stained with osmium, ferrous cyanide, lead acetate and uranyl acetate according to standard Autoslice and View protocol provided by FEI^7^. After sectioning, the polymer grided coverslip was dissolved in xylol for 1 h, and samples were mounted and sputter-coated with gold for 30 s using a Q150T ES coater (Quorum Technologies). Focused ion beam scanning electron microscopy (FIB-SEM) imaging was performed on a Helios NanoLabG3 microscope (FEI). Images were acquired at the highest resolution setting, resulting in 5×5×10 nm pixels using the Autoslice and View software (FEI). Drift correction and alignment were performed using Amira software. Confocal stacks were deconvolved using Imaris (Oxford Instruments). Orthogonal slices and overlay were generated from the aligned stack using ImageJ.

### Calcium imaging

Cells seeded on 25 mm coverslips were mounted on AttoFluor imaging chambers (Thermo), loaded with 4 μM Fura-2- AM, 0.01% pluronic (Thermo) in modified Ringer’s^5^ for 30 min at RT. Then, 340/380 nm excitation (ex) 510 ± 40 nm emission (em) ratiometric imaging was performed at 37 °C in modified Ringer’s (140 mM NaCl, 5 mM KCl, 1 mM MgCl_2_, 2 mM CaCl_2_, 20 mM HEPES, 10 mM glucose, pH 7.4) where Ca^2+^-free solution (CF) contained 1 mM EGTA instead of 2 mM CaCl_2_ (CA). Images were acquired using a widefield fluorescence Nikon Eclipse Ti microscope system (Visitron Systems) equipped with a 40X Plan Fluor 0.75 objective and stage heater. Frames were acquired every 3 s. Ca^2+^ microdomain/hotspot imaging^6^ was performed as follows: cells were loaded with 4 μM Fluo-8-AM (AAT Bioquest) for 30 min at 37 °C, 30 min at RT and 2.5 μM BAPTA-AM for the last 10 min, in modified Ringer’s/500 μM sulfinpyrazone. Simultaneous ex/em at 488/530 and 543/640 nm were collected in separate channels. Periphagosomal Ca^2+^ hotspots, images were averaged over 6 s and captured between 20 and 30 min after the addition of phagocytic targets. Image analysis was carried out with ImageJ on background-subtracted images according to prior protocols^5,6^, where hotspots were defined as 4 pixel areas (500 nm^2^) of Fluo-8 fluorescence at least two standard deviations higher than the average cytosolic mean fluorescence intensity, occurring within a distance of 3 pixels (750 nm) from the phagosomal border.

### TIRF imaging

Total internal reflection microscopy (TIRF) imaging was performed and quantified according to prior protocols^70^ where transfected cells were labeled with Cell Mask Deep Red (Thermo) diluted 1:1000 in 0.5 ml CA for 10 min, then rinsed 3× in 1 ml CF to define the TIRF focal plane. Experiments were carried out at 37 °C and imaged using a Nikon Eclipse Ti with a Perfect Focus system and 100×1.49 Oil CFI Apochromat TIRF objective at a rate of 1 frame/s. Quantification was performed with ImageJ and the Analyze particles plug-in, and Prism for curve-fitting and calculations. MCS puncta were defined on background-subtracted images as regions above threshold >4 pixels (500 nm^2^), where threshold was defined as whole-cell fluorescence plus 1 standard deviation. Cells with puncta >500 nm^2^ at baseline were excluded from the analysis.

### Phagosomal phospholipid assessment

Cells seeded on 25 mm coverslips and transfected with various lipid probes were mounted on AttoFluor chambers and washed in CA. Using a Nipkow Okagawa Nikon spinning-disk confocal imaging system equipped with a temperature control chamber, stage motor and Plan Apo 63x/1.4 Oil DICIII objective and Visiview software (Visitron Systems). IgG-coupled phagocytic targets were added, and 3–5 stage positions were selected near cells that began phagocytosing 5 min after bead addition. Confocal z-stacks of 9 frames spaced at 0.5 µm intervals were then acquired every 1 min at each stage position for 30–40 min. Images were taken in either 488/530, 561/630 nm ex/em or both depending on transfected probes. Background-subtracted maximum projections of confocal stacks were generated and initial total cell fluorescence as well as individual phagosome tracks analyzed using ImageJ. Phospholipid enrichment was defined as the phagosomal fluorescence divided by the initial total cell fluorescence minus 1 and computed using Excel (Microsoft).

### Phagosomal pH

Phagosomal pH was assessed by ratiometric imaging using previously established protocols^71^, with pH-sensitive opsonized fluorescein isothiocyanate (FITC)-coupled sRBCs employed as phagocytic targets. Transfected cells seeded on 25 mm coverslips were mounted in AttoFluor chambers, washed in medium, exposed to phagocytic targets, centrifuged at 200×*g* for 1 min and incubated at 37 °C. After 15 min, coverslips were washed 5x medium to remove unbound targets. At 35 min, cells were washed in CA, and 5 images at 440/530 and 480/530 nm ex/em were captured after 40, 60 and 90 min of target addition using the microscope as for Ca^2+^ imaging described above. Calibrations were performed on 5 separate stacks per solution, using nigericin (5 mg/ml)/monensin (5 μM) and potassium chloride buffers of pH 4–9 (140 KCl, 1 mM MgCl_2_, 20 mM NaCl, 0.2 mM EGTA, with 20 mM pH buffers: potassium citrate for pH 4.0–4.5, MES for pH 5.0–6.5, HEPES for pH 7.0–7.5, Tris for pH 8.0–9.0), where each solution was equilibrated for 3 min. Image analysis on background-subtracted images was performed with ImageJ, and pH calculations from calibration curves were computed using Prism and Excel.

### Phagolysosome fusion (PLF) assay

Phagolysosome fusion assays based on a previously published fluorescence resonance energy transfer (FRET) assay^7,72^ as follows. Transfected cells were loaded with 10 µg/ml AF594-hydrazide (AF594-HA, Thermo) overnight and chased for 3 h into lysosomes after rinsing 5x in full medium. AF488-IgG beads were added, centrifuged at 200×*g* for 1 min, incubated for 30 min, and washed to remove un-internalized beads. Images were recorded using the microscope as for Ca^2+^ imaging described above, except the 60x Plan-Apo 1.4 NA objective was used, and of 490/630 (FRET), 490/525 (green), 572/630 (red) nm ex/em, as well as brightfield channels were acquired. Stack of 15 planes spaced 0.8 µm in 5–10 fields per condition was recorded, and background-subtracted images were analyzed using ImageJ. PLF index was defined per cell as the phagosomal fluorescence in the FRET/green channel ratio divided by the sum total cell acceptor loading^7^.

### Immunoblotting

Cells were washed with cold PBS and resuspended in lysis buffer containing 25 mM Tris-HCl pH 7.6, 150 mM NaCl, 1% NP-40, 1% sodium deoxycholate, 0.1% SDS, in the presence of HaltProtease Inhibitor Cocktail, EDTA-Free (Thermo) and incubated on ice for 20 min. Lysates were sonicated for 10 min at 40 kHz in a bath sonicator (Emag Emmi-D280) and centrifuged at 18,000×*g* for 10 min at 4 °C. Total protein concentration was determined using Roti-Quant kit (Carl Roth) according to the manufacturer’s instructions. Lysates were diluted in Roti-Load (Carl Roth) and heated at 95 °C for 5 min. Then, 15–35 µg protein was loaded and separated by SDS-PAGE in 4–20% Mini-Protean TGX Precast gels (Bio-Rad). Gels were transferred to PVDF membranes using the iBlot system (Thermo). Membranes were blocked for 1 h in 5% low-fat powdered milk/1% Tween/TBS (TBS-T), washed and incubated overnight in primary antibodies at 4 °C and for 1 h at RT in HRP-conjugated secondary antibodies, all diluted in 3% milk/TBS-T. Signals were detected using Immobilon Western Chemiluminescent HRP Substrate (Millipore) and the ImageQuant LAS 4000 mini. Tubulin was used as an internal loading control, and the relative intensities of protein bands were quantified using ImageJ and Excel.

### Immunoprecipitation

Immunoprecipitation was performed using Chromotek GFP-trap agarose beads (Allele Biotech) according to the manufacturer’s protocol^65^. Transfected cells were washed in cold PBS and lysed with buffer 50 mM Tris, 120 mM NaCl, 40 mM HEPES, 0.5% digitonin, 0.5% CHAPS, (pH 7.36) supplemented with a protease inhibitor cocktail (Roche). The cell lysates were then incubated with the beads for 1 h at 4 °C in rotation. After extensive washes, the immunoprecipitated proteins bound to the beads were eluted in 2% SDS sample buffer and boiled for 1 min prior to SDS-PAGE in a 10% gel and immunoblotting as above.

### Statistics and reproducibility

Prism (GraphPad Software) was used to conduct all statistical analyses. The specific statistical analysis performed, with all relevant information, is provided below the full dataset listed in the Supplementary Data 1 file. Significant *p*-values are listed above the bars. All images shown are representative of at least 3 independent samples. All measurements shown in bar graphs were taken from distinct samples. Time course data with the time labeled on the *X*-axis represent repeated measures of the same set of samples across time. Commercial software used for image acquisition (microscope control): Electron microscopy: Autoslice and View (FEI) 3.0. Spinning-disk confocal/widefield microscopes: Visiview 4.0 (Visitron Systems). Confocal microscope: Zen 2010b version service pack1 (Zeiss). Total Internal Reflection microscope: NIS Elements 3.0. Electron microscopy Image analysis was performed with Amira 3.0 software. Fluorescence image analysis was performed using ImageJ 1.53d. (NIH) except for deconvolution, performed with Imaris 8 (Oxford Instruments). Please note software versions are approximate, as some have changed over the years over which the study took place. No sample size calculation was performed. A minimum of *n* = 3 independent biological experiments were performed, with generally 4–6 performed per condition depending on the complexity/feasibility of the experiment. Blinding and randomization were not performed. For microscopy data, cells exhibiting abnormal morphology (multinucleated, high vacuolation) or signs of death (blebbing) were excluded from the analysis. For microscopy data, a minimum of 5 replicate fields per coverslip are imaged. These may contain from one to tens of cells or one to hundreds of phagosomes, which, depending on what is being measured, are the replicates. These numbers are indicated in the figure legend.

### Reporting summary

Further information on research design is available in the Nature Portfolio Reporting Summary linked to this article.

### Supplementary information

Peer review file

Supplementary Figures

Description of Additional Supplementary Data

Supplementary Dataset 1

Reporting Summary

## Data Availability

Source data, as well as statistical analysis for all graphs, are provided in the Excel file Supplementary Data 1. Source images for representative Western blots shown in figures are provided in Supplementary Fig. S6. Full datasets, including all microscopy and Western blot source images, are available on the University of Geneva’s FAIR-compliant data repository Yareta under a CC BY 4.0 licence at the following 10.26037/yareta:d6bpvmpn4rfchkw6mduh3woq3u. Plasmids generated in this study are available on Addgene, as cited above, to be distributed subject to a standard Material’s Transfer Agreement.
