# Supplementary material for: Sec22b regulates phagosome maturation by promoting ORP8-mediated lipid exchange at endoplasmic reticulum-phagosome contact sites
Source: Commun Biol. 2023 Oct 4;6:1008. doi: 10.1038/s42003-023-05382-0 (PMC10550925; doi:10.1038/s42003-023-05382-0)
Supplement: Supplementary file 2 — Supplementary Figures [file 42003_2023_5382_MOESM2_ESM.pdf]

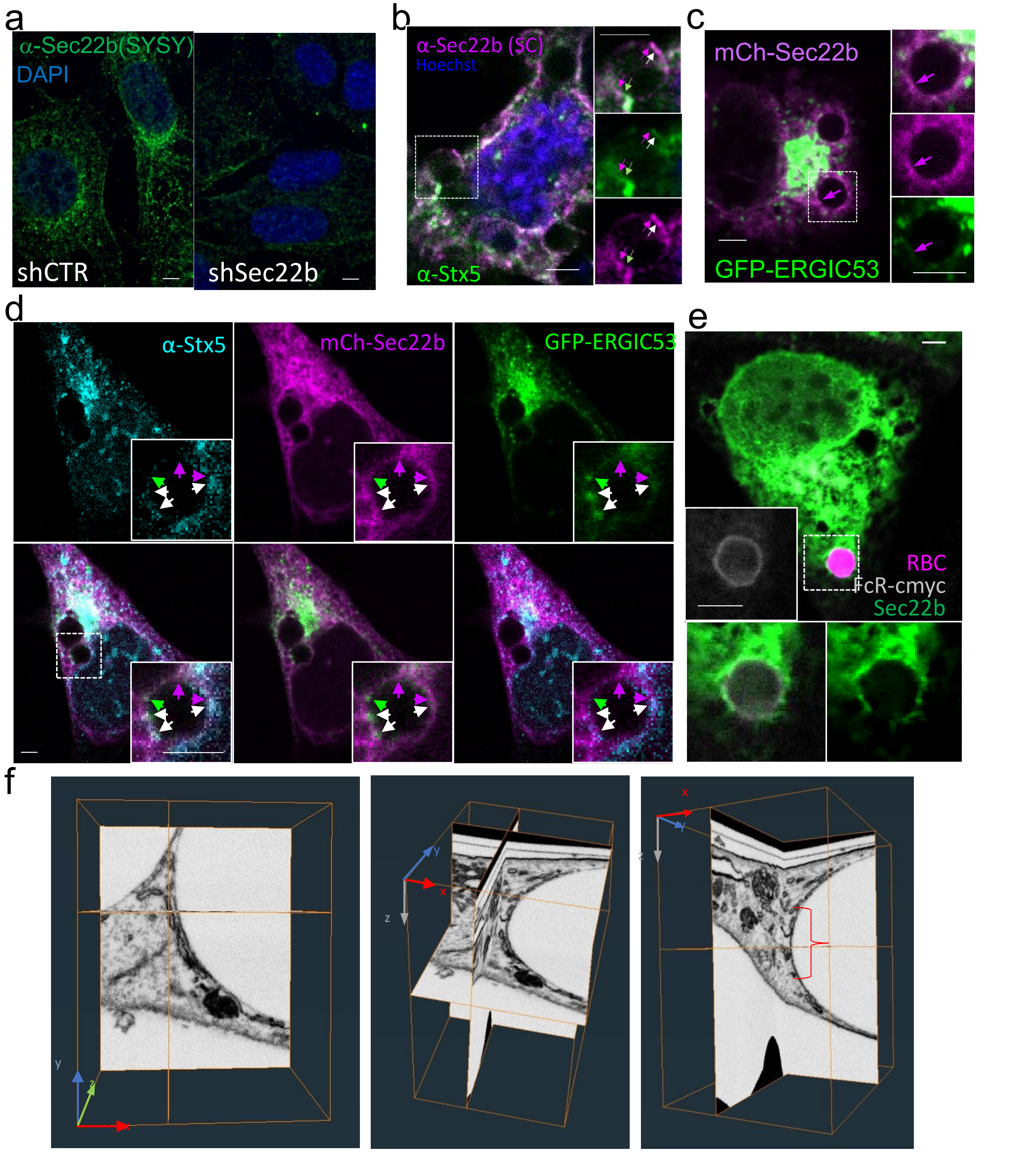

Figure S1. ***Related to Fig. 1. Sec22b localizes to ER-phagosome membrane contact sites.*** **a)** Immunostaining of MEFs stably transfected with control (shCTR) or Sec22b-targeting (shSec22b) shRNA with anti-Sec22b (SYSY, green) **b)** MEFs overexpressing FCGR2A(FcR)-c-myc (phagocytic MEFs) co-stained with anti-Stx5 (green) and anti-Sec22b (SC, magenta) after 30 min exposure to IgG-beads. Arrows show periphagosomal puncta positive for Sec22b (magenta), Stx5 (green) or both (white). **c)** Phagocytic MEFs co-transfected with mCh-Sec22b (magenta) and GFP-ERGIC-53 (green) after 30 min exposure to IgG beads. The arrow illustrates a periphagosomal structure positive for Sec22b (magenta) and devoid of the ERGIC marker (green). **d)** Cells as in c) but co-stained with anti-Stx5 (cyan). Arrows show periphagosomal puncta positive for Sec22b (magenta) or ERGIC-53 (green). White arrows show Stx5 positive periphagosomal puncta that co-localized with Sec22b, ERGIC-53 or both. **e)** Phagocytic MEFs expressing EGFP-Sec22b (green) stained to visualize Fc-receptors (white) and highlight the phagosomal membrane (with anti-cmyc) to distinguish it from periphagosomal puncta. Cells were exposed to pHrodo-labelled IgG-sheep red blood cells (sRBCs, magenta) for 15 min. White bars = 3 μm. **f)** The three panels show a sequential rotation (achieved by dragging the lower left-hand corner up) of orthogonal slices of the inset of the 3D EM stack shown in Fig. 1f. Red bracket in right panel depicts the area above and below the confocal plane examined for the presence of vesicles.

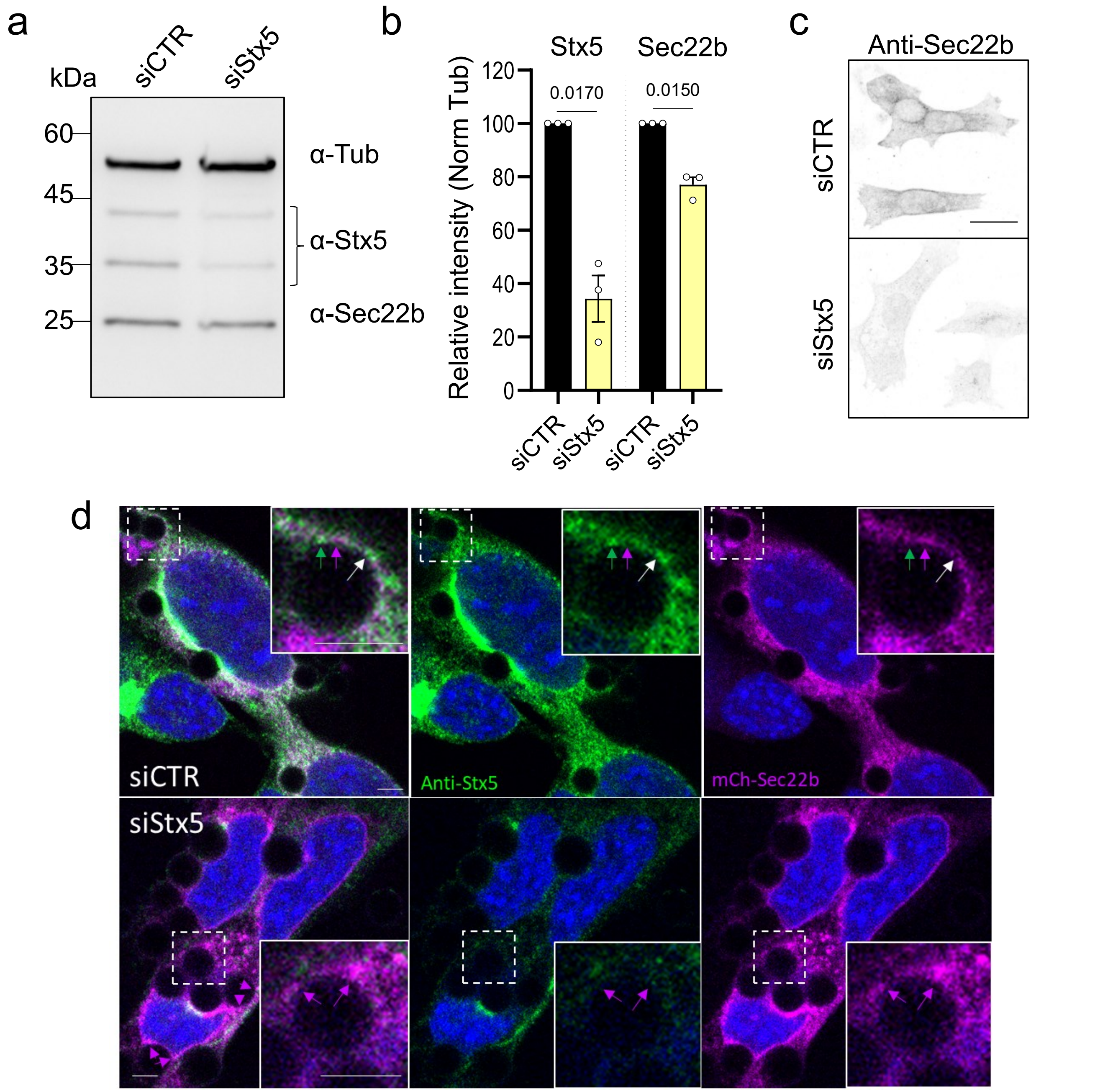

Figure S2. ***Related to Fig. 2. Western blot and immunostaining of Stx5 downregulation.*** **a)** Western blot of MEFs transfected with siCTR or siStx5 (10 nM) and sequentially blotted with anti-Stx5, anti-Sec22b (SYSY) and anti-tubulin (Tub) as loading control. **b)** Quantification of the sum of the two Stx5 bands corresponding to the long and short isoforms, or the Sec22b band. Values are normalized to the tubulin loading control and expressed as percentage of the paired siCTR sample (n=3 independent biological samples). **c)** Immunostaining of MEFs transfected with siCTR or siStx5 using anti-Sec22b(SYSY). An inversed linear grayscale lookup table (identical imaging and contrast settings) is used to make differences in Sec22b expression levels more visible. Bar = 10 μm. **d)** Phagocytic MEFs transfected with mCh-Sec22b and exposed to IgG-beads for 30 min. Arrows show periphagosomal puncta positive for Sec22b (magenta), Stx5 (green) or both (white). Bars = 3 μm. Bar graph show means +/- SEM.

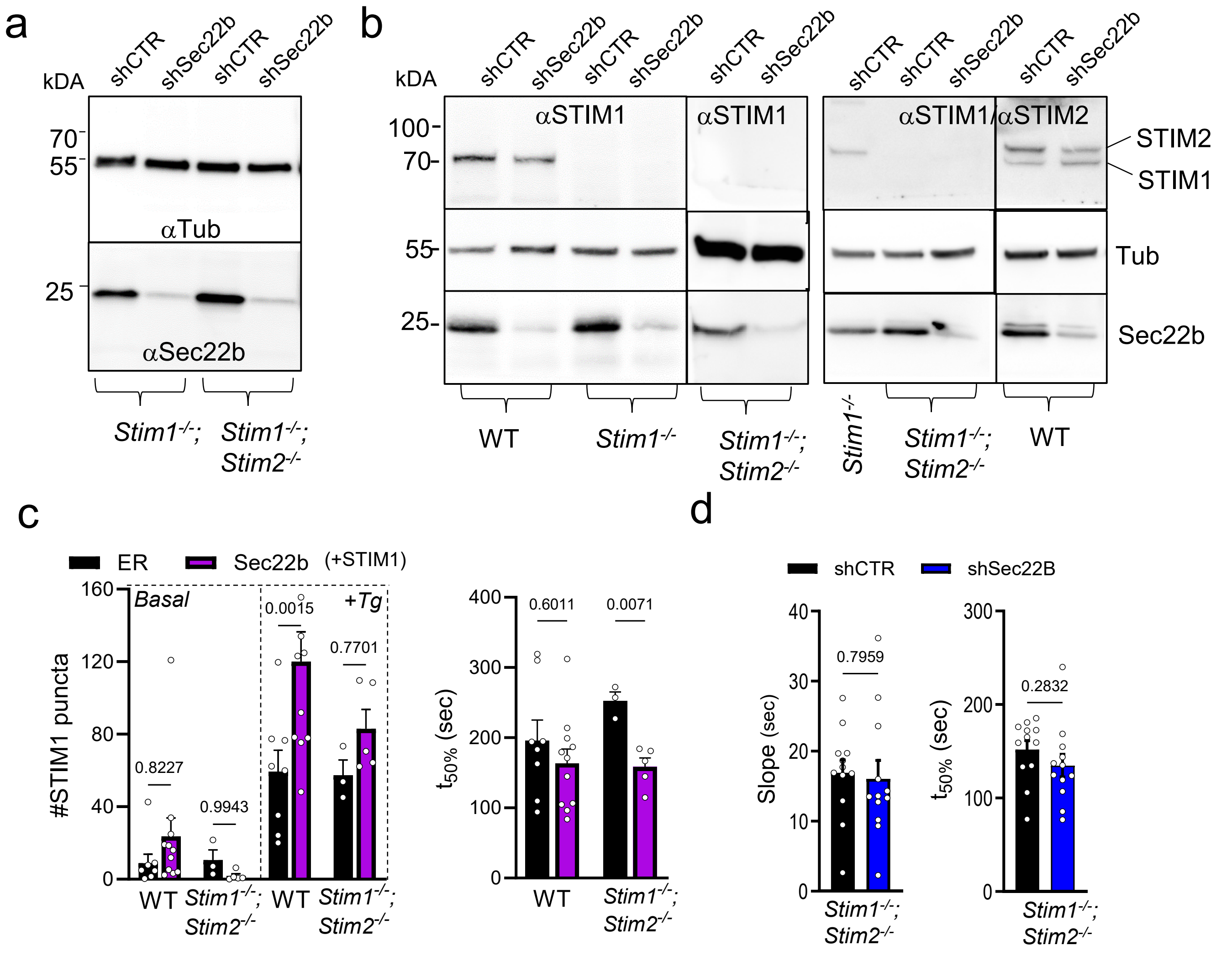

Figure S3. ***Related to Figs 3 and 4. Western blot and supplementary TIRF quantification.*** **a)** Western blots of *Stim1*$^{-/-}$ and *Stim1*$^{-/-}$*; Stim2*$^{-/-}$ MEF cell lines stably expressing shCTR and shSec22b incubated with anti-Sec22b (SYSY) and anti-tubulin (Tub) as control (quantification in Fig. 4e). **b)** Western blots of WT, *Stim1*$^{-/-}$ and *Stim1*$^{-/-}$*; Stim2*$^{-/-}$ stable cell lines incubated as above and with anti-STIM1 or anti-STIM1 and STIM2 as indicated, quantification in Fig. 4d). **c)** Related to Fig. 3a-c, quantification of the puncta number and kinetics, $t_{50\%}$ parameter of the Boltzman sigmoidal curve fit) of STIM1 puncta appearing at the TIRF plane after addition of Tg. (n=8/11;3/5 independent biological samples WT;*Stim1*$^{-/-}$;*Stim2*$^{-/-}$ KDEL/Sec22b, 14/15; 9/13 cells;) **d)** Quantification of the kinetics (slope and $t_{50\%}$ parameters of Boltzmann sigmoidal curve fits) of STIM1 puncta arrival at the TIRF plane in response to Tg. (n=13/12 independent biological samples shCTR/shSec22b, 22/19 cells). Related to Fig. 3d. Bar graphs show means +SEM

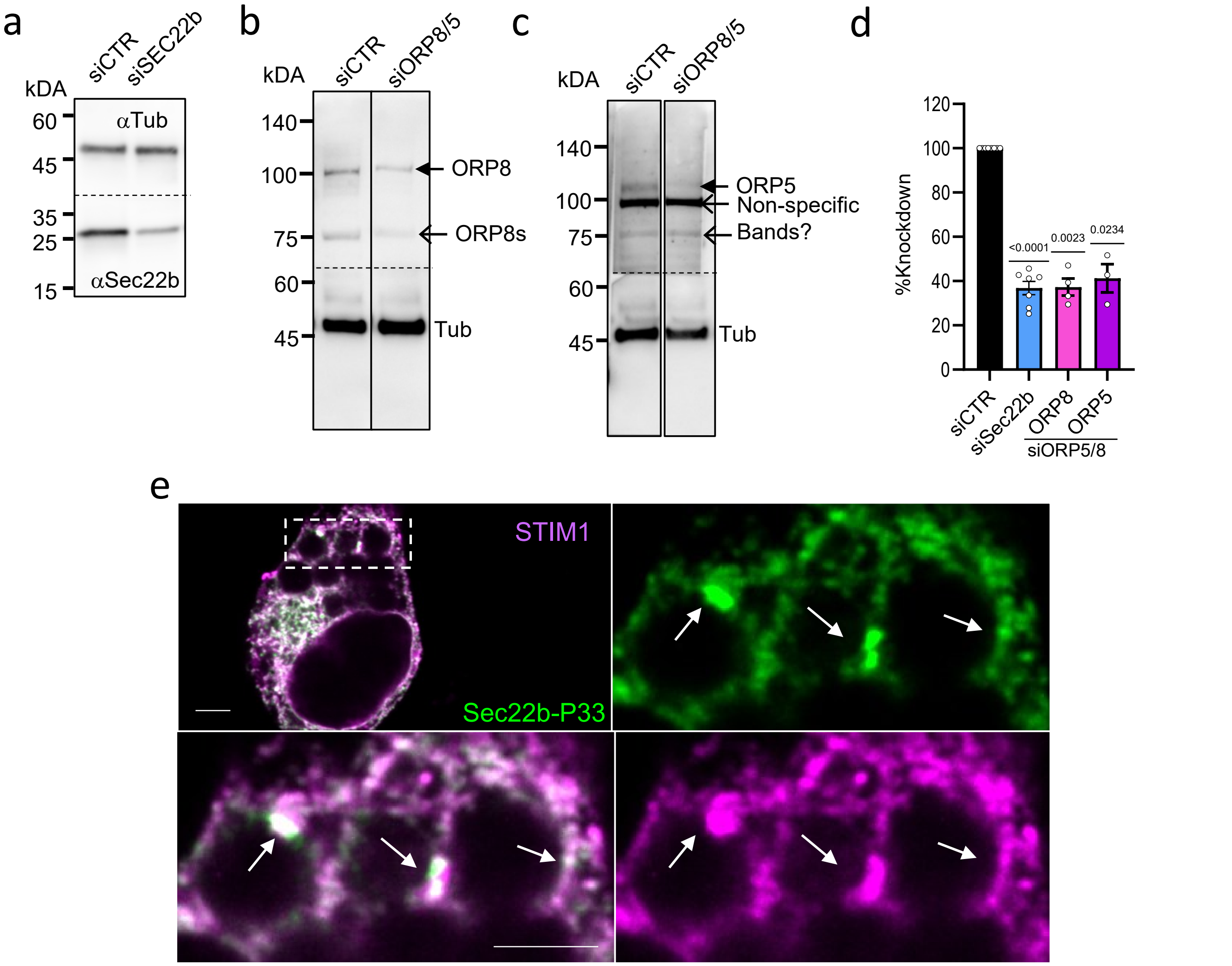

Figure S4. ***Related to Figs 5 and 6. Western blots for siRNA downregulation and Sec22b-P33 recruitment to phagosomes.*** ***a*****)** Western blots of WT MEFs transfected with siCTR or siSec22b (50 nM) for 48 h incubated with anti-Sec22b (SYSY) and anti-tubulin (Tub) as control (related to Figs. 5e and 6d). **b-c)** Western blots of WT MEFs transfected with siCTR (100 nM) or siORP5/8 (50+50 nM) for 48 h incubated with anti-ORP8 **(b)** or and anti-ORP5 **(c)** and anti-Tub as control (related to Fig. 5c). In (**b**) the two bands correspond roughly to the molecular weight of the canonical (100 kDa, closed arrow) and short (80 kDa) isoform (ORP8s, open arrow). In (**c**) the band corresponding to the typical running molecular weight of the canonical ORP5 isoform (110 kDa, closed arrow) responded to siRNA treatment while two other bands (presumed non-specific) did not. **d)** Quantification of Western blots sets represented in a-c for Sec22b and canonical ORP isoforms (n=8;7;4;3 independent biological samples for siCTR;siSec22b;ORP8 in siORP5/8; ORP5 in siORP5/8). **e)** Confocal images of mCh-STIM1 (magenta) and shR-EGFP-Sec22b-P33 (green) in shSec22b MEFs exposed to IgG-beads for 30 min (related to Fig. 6f). Arrows: periphagosomal puncta reminiscent of MCS showing overlap of STIM1 and P33. White bar = 3 µm. Bar graphs are means +/- SEM.

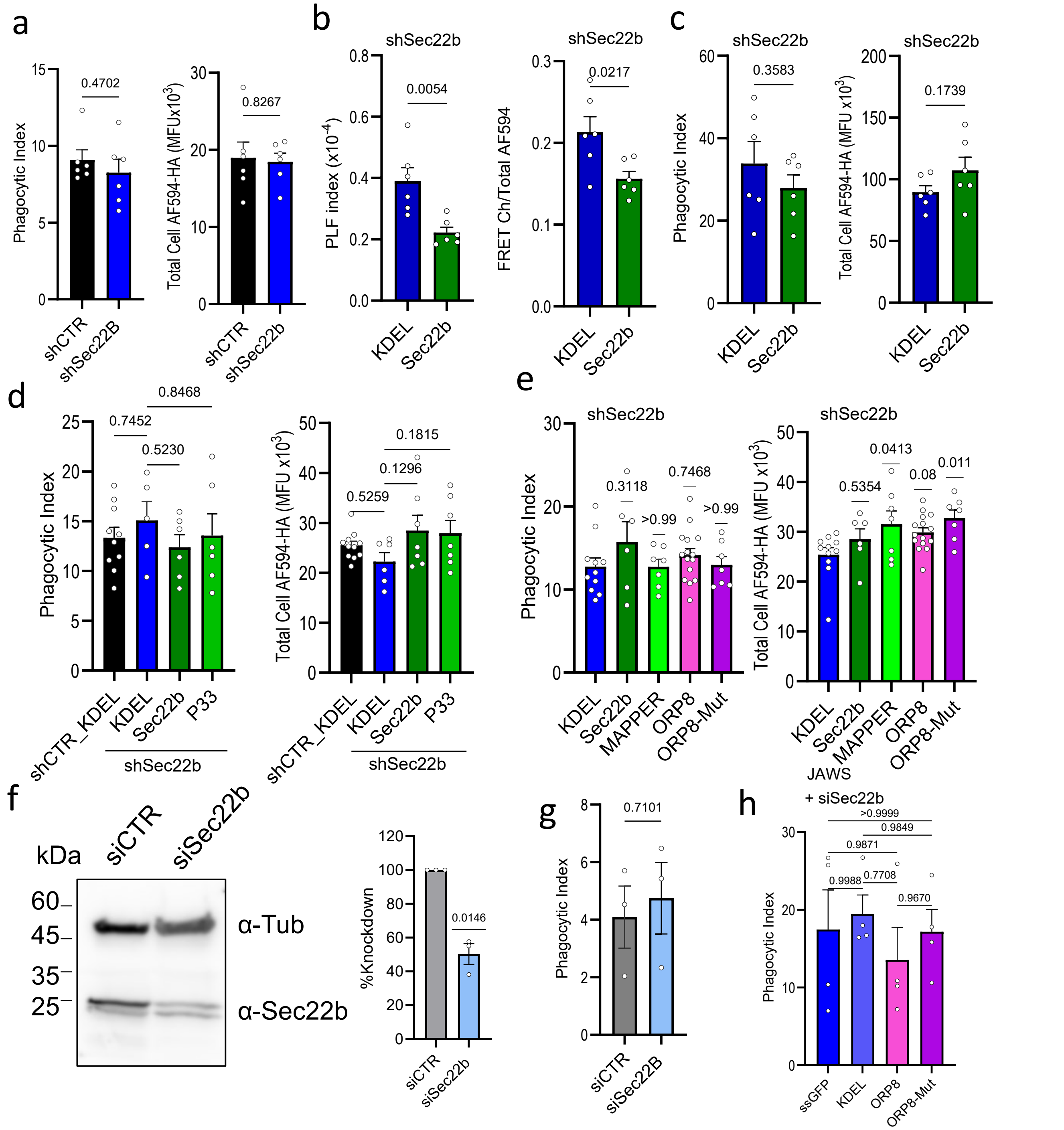

Figure S5. ***Related to Figure 7. Phagolysosome fusion and antigen degradation supporting material.*** **a)** Quantification of the number of phagosomes per cell (phagocytic index) and total cell loading of AF549-HA of shCTR and shSec22b cells analysed in Fig. 7a. (n= 6/6 independent biological samples, 1071/1163 phagosomes shCTR/shSec22b). **b)** PLF index measured as in Fig. 6 in phagocytic shSec22b MEFs transfected with GFP-KDEL or shR-EGFP-Sec22b, except cells were exposed at 1:50 cells:AF488-IgG-bead ratio. FRET Ch/Total AF594 (FRET channel normalized to total cell acceptor loading) is an alternate calculation of PLF index that omits the GFP/AF488 donor channel normalization. (n=6/6 independent biological samples, 3889/2304 phagosomes). **c)** Quantification of phagocytic index and total cell loading of AF549-HA of the cells analysed in (**b**). **d)** Quantification of the phagocytic index and total cell loading of AF549-HA of the cells analysed in (Fig 7**c**). **e)** Quantification of the phagocytic index and total cell loading of AF549-HA of the cells analysed in Fig. 7d. **f)** Western blots of JAWS cells transfected with siCTR or siSec22b (50 nM) for 24 h and incubated with anti-Sec22b (SYSY) and anti-tubulin (Tub) as control (related to Fig. 7e) (n=3/3 independent biological samples). **g)** Quantification of the phagocytic index of cells analysed in Fig. 7g. **h)** Quantification of the phagocytic index of cells analysed in Fig. 7h. Bar graphs are means + SEM

Original Western Blot Gel Images

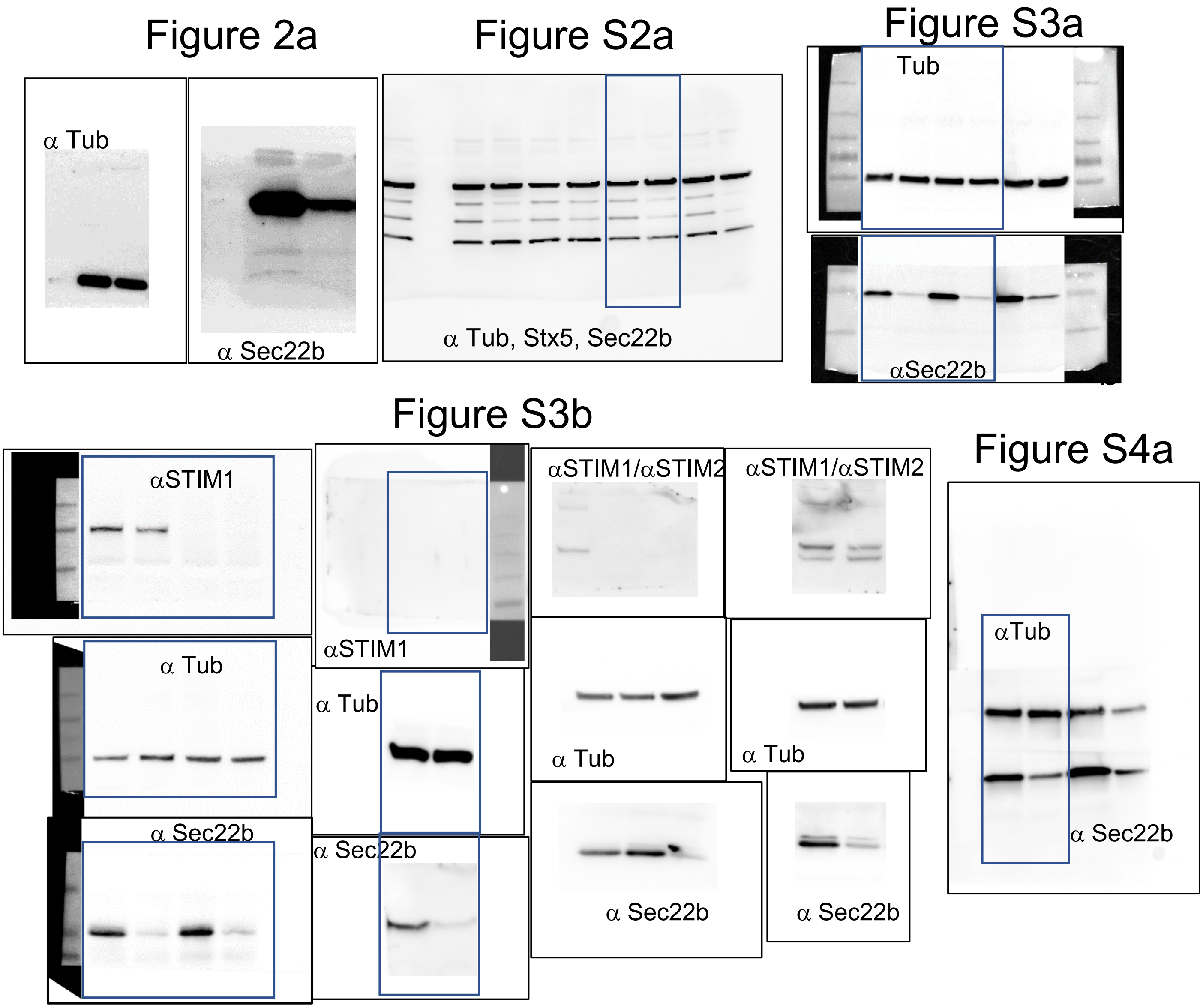

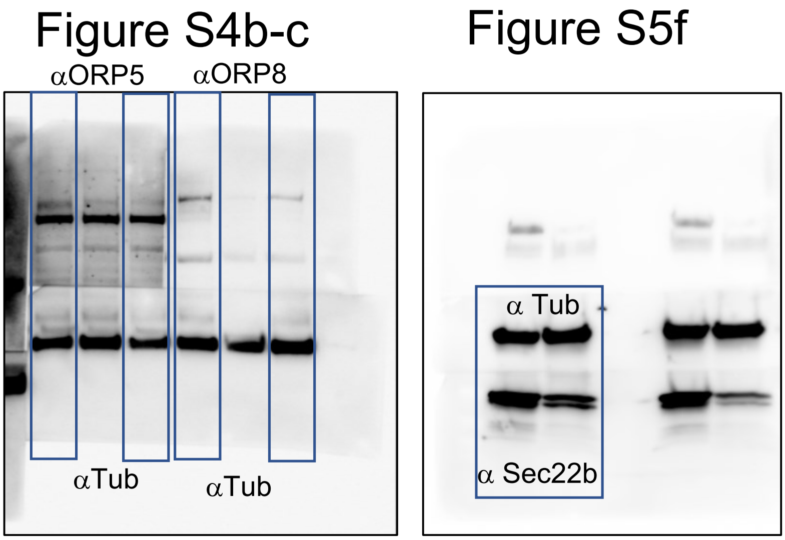

Figure S6. *Original images of representative blots shown in Figures 2a, S2a, S3a-b, S4a-c and S5f.* *Highest exposure available shown for all blots. The lanes shown in the figures are highlighted in a blue box. Please note that membranes were cut prior to blotting for all blots except that of Fig S2a.*
