# Supplementary material for: Sec22b regulates phagosome maturation by promoting ORP8-mediated lipid exchange at endoplasmic reticulum-phagosome contact sites
Source: Commun Biol. 2023 Oct 4;6:1008. doi: 10.1038/s42003-023-05382-0 (PMC10550925; doi:10.1038/s42003-023-05382-0)
Supplement: Supplementary file 5 — Reporting Summary [file 42003_2023_5382_MOESM5_ESM.pdf]

nature portfolio

Corresponding author(s): Paula Nunes-Hasler

Last updated by author(s): Sep 18, 2023

Reporting Summary

Nature Portfolio wishes to improve the reproducibility of the work that we publish. This form provides structure for consistency and transparency in reporting. For further information on Nature Portfolio policies, see our Editorial Policies and the Editorial Policy Checklist.

Statistics

For all statistical analyses, confirm that the following items are present in the figure legend, table legend, main text, or Methods section.

n/a	Confirmed	
☐	☒	The exact sample size (*n*) for each experimental group/condition, given as a discrete number and unit of measurement
☐	☒	A statement on whether measurements were taken from distinct samples or whether the same sample was measured repeatedly
☐	☒	The statistical test(s) used AND whether they are one- or two-sided<br>*Only common tests should be described solely by name; describe more complex techniques in the Methods section.*
☒	☐	A description of all covariates tested
☐	☒	A description of any assumptions or corrections, such as tests of normality and adjustment for multiple comparisons
☐	☒	A full description of the statistical parameters including central tendency (e.g. means) or other basic estimates (e.g. regression coefficient) AND variation (e.g. standard deviation) or associated estimates of uncertainty (e.g. confidence intervals)
☐	☒	For null hypothesis testing, the test statistic (e.g. *F*, *t*, *r*) with confidence intervals, effect sizes, degrees of freedom and *P* value noted<br>*Give P values as exact values whenever suitable.*
☒	☐	For Bayesian analysis, information on the choice of priors and Markov chain Monte Carlo settings
☒	☐	For hierarchical and complex designs, identification of the appropriate level for tests and full reporting of outcomes
☒	☐	Estimates of effect sizes (e.g. Cohen's *d*, Pearson's *r*), indicating how they were calculated

Our web collection on statistics for biologists contains articles on many of the points above.

Software and code

Policy information about availability of computer code

Data collection	Commercial software used for image acquisition (microscope control): Electron microscopy: Autoslice and View (FEI) 3.0. Spinning disk confocal/widefield microscopes: Visiview 4.0 (Visitron Systems). Confocal microscope: Zen 2010b version service pack1 (Zeiss). Total Internal Reflection microscope: NIS Elements 3.0. Please note software versions are approximate, as some have changed over the years over which the study took place.
Data analysis	Electron microscopy Image analysis was performed with Amira 3.0 software. Fluorescence image analysis was performed using ImageJ 1.53d. (NIH) except for deconvolution, performed with Imaris 8 (Oxford Instruments). Please note software versions are approximate, as some have changed over the years over which the study took place.

For manuscripts utilizing custom algorithms or software that are central to the research but not yet described in published literature, software must be made available to editors and reviewers. We strongly encourage code deposition in a community repository (e.g. GitHub). See the Nature Portfolio guidelines for submitting code & software for further information.

Data

Policy information about availability of data

All manuscripts must include a data availability statement. This statement should provide the following information, where applicable:

- Accession codes, unique identifiers, or web links for publicly available datasets
- A description of any restrictions on data availability
- For clinical datasets or third party data, please ensure that the statement adheres to our policy

All data will be fully accessible under a CC-BY 4.0 license on the University of Geneva's data repository Yareta https://doi.org/10.26037/yareta:d6bpvmpn4rfchkw6mduh3woq3u. Plasmids generated in this study are made available on Addgene subject to a standard Material Transfer Agreement.

Human research participants

Policy information about studies involving human research participants and Sex and Gender in Research.

Reporting on sex and gender	There is no human research in this study
Population characteristics	There is no human research in this study
Recruitment	There is no human research in this study
Ethics oversight	There is no human research in this study

Note that full information on the approval of the study protocol must also be provided in the manuscript.

Field-specific reporting

Please select the one below that is the best fit for your research. If you are not sure, read the appropriate sections before making your selection.

☒ Life sciences ☐ Behavioural & social sciences ☐ Ecological, evolutionary & environmental sciences

For a reference copy of the document with all sections, see nature.com/documents/nr-reporting-summary-flat.pdf

Life sciences study design

All studies must disclose on these points even when the disclosure is negative.

Sample size	No sample size calculation was performed. As a rule of thumb, in the field for in vitro studies such as this on a minimum of n=3 is commonly accepted, we generally used 4-6 depending on the complexity/feasibility of the experiment.
Data exclusions	For microscopy data, cells exhibiting abnormal morphology (multinucleated, high vacuolation) or signs of death (blebbing) were excluded from analysis
Replication	For microscopy data a minimum of 5 replicate fields per coverslip are imaged. These may contain from 1 to tens of cells or 1 to hundredes of phagosomes which, depending on what is being measured, are the replicates. These number are indicated in the figure legends.
Randomization	Randomization was not used (in vitro study)
Blinding	Blinding was not used as in most cases it was not possible (groups were obvious)

Reporting for specific materials, systems and methods

We require information from authors about some types of materials, experimental systems and methods used in many studies. Here, indicate whether each material, system or method listed is relevant to your study. If you are not sure if a list item applies to your research, read the appropriate section before selecting a response.

Materials & experimental systems

n/a	Involved in the study
☐	☒ Antibodies
☐	☒ Eukaryotic cell lines
☒	☐ Palaeontology and archaeology
☒	☐ Animals and other organisms
☒	☐ Clinical data
☒	☐ Dual use research of concern

Methods

n/a	Involved in the study
☒	☐ ChIP-seq
☒	☐ Flow cytometry
☒	☐ MRI-based neuroimaging

Antibodies

Antibodies used

The following antibodies (antibody name/catalog#/dilution, IF: immunofluorescence, WB: Western blot) were purchased from: Synaptic Systems (SYSY): rabbit anti-Sec22b (186003/1:200 IF, 1:1000 WB), rabbit anti-Stx5 (110053/1:100 IF, 1:1000 WB); Santa Cruz: mouse anti-Sec22b (29-F7) (sc-101267/1:100); Cell Signaling: mouse anti-c-myc antibody (9B11) (2276/1:100), rabbit anti-STIM2 (4917S/1:1000); Thermo: mouse anti-CD16-CD32 (Fc-Block, 14-0161-85, 1:200), rabbit anti-ORP5 (PA5-18221/1:500), goat-anti-rabbit Alexa Fluor 555 (A21428/1:1000), goat-anti-mouse Alexa Fluor 647 (A21235/1:1000), goat-anti-human Alexa Fluor 633 (A-21091/1:500); GeneTex: rabbit anti-ORP8 (GTX121273/1:500); Sigma: mouse anti-α-tubulin (T9026/1:5000), rabbit anti-sheep red blood cell (sRBC, S1389/1:40), mouse anti-FLAG-M2 (F1804/1:1000); mouse anti-GFP (11814460001/1:1000); BD Biosciences: mouse anti-GOK/STIM1 (610954/1:100); ABCD Antibodies: human IgG1-anti-EPEA (AI215-H1/1:100); Bio-Rad: goat anti-rabbit IgG (H+L) HRP conjugate (170-6515/1:10000), goat anti-mouse IgG (H+L) HRP conjugate (170-6516/1:10000), Innovative Research: human IgG protein A purified (hIgG) (IR-HU-GF); Jackson ImmunoResearch: donkey-anti-mouse Alexa Fluor 488 (715-545-150/1:800), goat-anti-rabbit Alexa Fluor 647 (111-605-003/1:1000), goat-anti-rabbit DyLight 405 (111-475-003/1:800).

Validation

The Sec22b, Stx5, ORP8, ORP5, STIM1 and STIM2 antibodies are validated by knockdowns in the study.

Eukaryotic cell lines

Policy information about cell lines and Sex and Gender in Research

Cell line source(s)

MEFs, (ATCC CRL-2991), JAWSII DCs (ATCC CRL-11904), HeLa cells from ECACC (93021013). Stim1-/- cells were obtained from Dr. Michalak (see methods). Stim1-/-;Stim2-/- were obtained from Dr. Oh-hora (see methods)

Authentication

JAWSII were directly purchased. Hela cells were authenticated by genomic profiling (STRs). The other cell lines were not authenticated.

Mycoplasma contamination

All cell lines were tested every 6-12 months either by PCR or Mycostrips and were negative.

Commonly misidentified lines
(See ICLAC register)

Hela cells are listed as commonly misidentified.
