# Supplementary material for: Sec22b regulates phagosome maturation by promoting ORP8-mediated lipid exchange at endoplasmic reticulum-phagosome contact sites
Source: Commun Biol. 2023 Oct 4;6:1008. doi: 10.1038/s42003-023-05382-0 (PMC10550925; doi:10.1038/s42003-023-05382-0)
Supplement: Supplementary file 1 — Peer review file [file 42003_2023_5382_MOESM1_ESM.docx]

Peer Review File

Reviewers' comments:

Reviewer #1 (Remarks to the Author):

In this study, Criado Santos and collaborators have investigated the mechanisms through which the SNARE Sec22b regulates phagosome maturation. To this end, they used complementary cell biology approaches to understand the relationship between Sec22b and membrane contact sites (MCS) at the ER-phagosome interface. This study was performed in mouse embryonic fibroblasts expressing FcgammaR2A. The major finding of that study is that Sec22b affects a number of parameters associated to phagosome maturation by acting as a theter at ER-phagosome MCS. In particular, Sec22b was shown to mediate phagosomal phospholipid dynamics. Overall, this a very well performed study. The results are clearly presented and appropriate controls were included.

However, to further support the notion that interaction of Sec22b with ER-phagosome MCS is not related to fusogenic pathways, the authors should assess the localization of the Sec22b cognate ER SNARE Stx5 with respect to MCS and to assess the impact of Stx5 KD on Sec22b-MCS interactions (including phagosomal phospholipid dynamics).

Reviewer #2 (Remarks to the Author):

The manuscript by Santos et al describes findings on the dynamics and role of SNARE Sec22b in phagocytosis and phagosome-ER contacts, examining impact on global and local Ca2+ signalling and phagosome maturation. The authors employed an engineered phagocytic system whereby MEFs or other cells ectopically express FcgR2A, which was converts these cells into phagocytes of IgG-coated particles. They then observed the dynamics and distribution of ER, STIM, and Sec22b around phagosomes finding that Sec22b formed punctate structures that partially colocalized with STIMs, and enhanced contact sites by immunofluorescence, live-cell imaging, and CLEM. They found that Sec22b overexpression and silencing could modulate global Ca2+ signalling, but did not have much impact on local Ca2+ signalling during phagosome maturation. Instead, manipulation of Sec22b affected phophoinositide dynamics on phagosomes. For example, silencing of Sec22b delayed loss of PI4P on phagosomes relative to control cells, while PI3P was eliminated more quickly. They found Sec22b contacts may aid in lipid exchange between ER-phagosomes (PI4P vs PS) as supported by reduction in PS on phagosomes in Sec22b-silenced cells and that this may be mediated by ORP8. Ultimately, the presence of Sec22b and formation of contacts may affect rate of phagosome-lysosome fusion. This is supported by Sec22b silencing causing enhanced phagosome-lysosome fusion as judged by increased delivery of lysosome-loaded FRET donor onto phagosomes with FRET acceptor. The authors propose in the discussion that this may have consequences for processes like antigen processing, which is an interesting idea about the role of phagosome-ER contacts. This is a very interesting manuscript that expands on the ever-growing field of organelle contact sites and better positions about the role of phagosome-ER contacts in modulating phagosome maturation and function. I favour publication in Communications Biology, if the authors address my concerns below – some editorially, some experimentally.


1. The most significant issue with this work is that the findings are all strictly done on engineered phagocytes. While I agree that this system is more facile than manipulating macrophage cell lines, it is no longer the case that this is impossible as judged by the many studies using macrophage cell lines like RAW, J744, etc and even primary macrophages. The results and data are exceptional, but the authors must select key experiments and see if the results hold or are different in common cell lines such as RAW macrophages. I would suggest transfecting Sec22b in RAWs to follow dynamics on phagosomes, silencing of Sec22b to follow dynamics of PI4P on phagosomes, and silencing of Sec22b on phagosome-lysosome fusion.

2. While I very much think that contacts are being formed between ER-phagosomes, I think phagosome fractionation and probing for Sec22b on isolated phagosomes would boost the confidence of their conclusions, especially on actual macrophages. I would expect that ER-phagosome contacts will survive the fractionation.

3. The finding that Sec22b silencing increases phagosome-lysosome fusion is critical. Yet, the findings are solely based on FRET by delivery a lysosome-donor to a phagosome-acceptor. This is a cool technique. However, while authors also examined pH, I think this finding is too important to rely only on this and again on an engineered phagocyte. In fact, the authors spend a bit of time speaking to possible issues with FRET and addressing these. So, the authors must complement their phagosome-lysosome fusion assays by i) immunostaining with LAMP1 or other lysososmal markers (though this is also found on late endosomes, so it may be different) and/or ii) preload lysosomes with an fluorescent endocytic tracer like dextran/lucifer yellow and look for fusion or lack with phagasomes. Moreover, and rather importantly given their discussion, they should assess the proteolytic environment of phagosomes – Magic Red would be the easiest approach This should be done in both their phagocytic model and RAW cells.

4. It is not always clear if data are from at least three independent experiments, and/or how many cells or phagosomes were examined. Please add these details to the respective figure legend. It is not clear if data are being analysed for the means of independent experiments (appropriate in most cases) vs. all data points (rare depending on design). Please review SuperPlots review published in JCB (PMID 32346721). I strongly urge the authors to disclose most of their data as scatter/violin plots instead of column graphs.

Minor:
1. There are some grammatical/spelling issues that need to be addressed. For example, the lipids should be phosphatidylinositol-3-phosphate, NOT phosphatidylinositide.

2. The sentence in the abstract that starts with “Concordantly..” is confusing because I don’t know what is being knockdown and there is not enough context in the abstract to appreciate what the authors are getting at here.

3. DKO is used often, but I find this confusing. Refer to the actual deletion.

Reviewer #2 (Remarks to the Author):

I think the authors have addressed most of my concerns appropriately, either by experimentation or editorially. I accept that in some of their attempts to address remaining issues, that they faced technical difficulties. I do not think that these affect the overall story and their interpretation. Overall, I think there is significant value in publishing this work as revised that outway any remaining issues. I recommend publication now.

**Open Access** This file is licensed under a Creative Commons Attribution 4.0 International License, which permits use, sharing, adaptation, distribution and reproduction in any medium or format, as long as you give appropriate credit to

the original author(s) and the source, provide a link to the Creative Commons license, and indicate if changes were made. In the cases where the authors are anonymous, such as is the case for the reports of anonymous peer reviewers, author attribution should be to 'Anonymous Referee' followed by a clear attribution to the source work. The images or other third party material in this file are included in the article’s Creative Commons license, unless indicated otherwise in a credit line to the material. If material is not included in the article’s Creative Commons license and your intended use is not permitted by statutory regulation or exceeds the permitted use, you will need to obtain permission directly from the copyright holder. To view a copy of this license, visit http://creativecommons.org/licenses/by/4.0/.
